# Current physical therapy practice and implementation factors regarding the evidence-based ‘Rehabilitation of Mobility after Stroke (ReMoS)’ guideline in Germany: a cross-sectional online survey

**DOI:** 10.1186/s12883-022-02780-5

**Published:** 2022-07-30

**Authors:** Bettina Scheffler, Florian Schimböck, Almut Schöler, Katrin Rösner, Jacob Spallek, Christian Kopkow

**Affiliations:** 1 Department of Therapy Sciences I, Brandenburg University of Technology Cottbus-Senftenberg, Universitaetsplatz 1, 01968 Senftenberg, Germany; 2 Department of Nursing Sciences and Clinical Nursing, Brandenburg University of Technology Cottbus-Senftenberg, Senftenberg, Germany; 3 Institute of Health Sciences, Department of Physiotherapy, University of Luebeck, Lübeck, Germany; 4 Department of Public Health, Brandenburg University of Technology Cottbus-Senftenberg, Senftenberg, Germany

**Keywords:** Stroke, Rehabilitation, Physical therapy, Guideline adherence, Implementation factors, Cross-sectional study, Online survey

## Abstract

**Background:**

Evaluation of the current physical therapy practice for German stroke rehabilitation with respect to the ‘Rehabilitation of Mobility after Stroke (ReMoS)’ guideline recommendations and the associated implementation factors.

**Methods:**

A descriptive cross-sectional study employing an online survey was performed among German physical therapists in 2019. The survey consisted of three sections with open and closed questions: 1) self-reported use of ReMoS recommendations, 2) barriers of guideline use and 3) socio-demographic characteristics. The benchmark level for guideline adherent physical therapy was set at > 80%.

**Results:**

Data from 170 questionnaires were eligible for analysis. Participants’ mean age was 41.6 years, 69.4% were female, while 60.1% had no academic degree. The ReMoS guideline was unknown to 52.9% of the responders. Out of all the 46 ReMoS guideline recommendations, only ‘intensive walking training without a treadmill’ was reported to be performed in a guideline adherent manner. Respondents usually denied any personal limitations, such as limited knowledge, or that the ReMoS guideline did not fit their routine practice.

**Conclusions:**

Among German physical therapists, the ReMoS guideline is not well-known and many interventions are not performed as recommended, illustrating the discrepancies between the ReMoS guideline recommendations and current physical therapy practice. Interventions aimed at overcoming this gap should consider both knowledge of existing barriers and facilitators of guideline usage.

**Trial registration:**

The study was retrospectively registered to the German Clinical Trials Register (DRKS00026681).

**Supplementary Information:**

The online version contains supplementary material available at 10.1186/s12883-022-02780-5.

## Background

Stroke is a leading cause of death and disability worldwide, accounting for approximately 11.6% of deaths and 143 million disability-adjusted life years [1]. Due to improved survival rates and in view of the progressive ageing of the population, Wafa et al. 2020 predict that the number of stroke patients in Europe will increase by 27% by the year 2047 [2]. In the adult population in Germany, 2.5% of all people have already experienced a first-time stroke, with about 200,000 new cases arising every year [3].

People who have suffered a stroke are 65–121% more likely to rely on personal assistance or aids for self-care (e.g. for bathing), mobility (e.g. for standing and walking) and household activities than people who have not been affected by a stroke [4]. Regaining motor function and mobility is of great importance for stroke survivors [5, 6]; as it is associated with a higher likelihood of being discharged home after an initial hospital treatment [7], as well as a higher health-related quality of life and community integration in the medium term [8]. In contrast to other functional systems, motor function shows the greatest increase during the first three months after the infarction [9]. Yet, as functional improvement after stroke is most likely to be incomplete, only up to 53% will restore their ability to walk outdoors without supervision [10–12].

A multi-disciplinary rehabilitation has been proven to be effective regarding the improvement of motor skills and mobility after a stroke [13]. In Germany, 54.4% patients receive rehabilitation measures after suffering a stroke [14]. Depending on the treatment and rehabilitation goals, the tasks and services of rehabilitation providers are structured into phases A-F according to the phase model established by the Federation of German Pension Insurance Institutions in 1994. After acute care (phase A), when patients might still require intensive medical treatment and independent activities of daily living are still not possible, activating care and targeted functional treatment is commenced for several hours per day by more than one therapist at the same time (phase B). After completion of early rehabilitation in phase B, patients usually continue rehabilitation in an inpatient facility in order to regain independence in their basic activities of daily living (phase C and D) or, if rehabilitation options are limited, are discharged directly to a long-term care facility (phase F). In rehabilitation phase E, an independent, self-determined life within the social and professional community can be aimed for, with therapeutic measures being carried out in an outpatient setting. Nevertheless, patients who have suffered stroke do not necessarily go through all these phases chronologically, but rather according to regular evaluation and reorientation oriented towards individual needs [15].

Physiotherapists who practice in Germany for stroke rehabilitation are usually trained at vocational schools [16, 17]. The content of such professional education and the examination methods are based on the Training and Examination Ordinance for Physiotherapists (PhysTh-AprV) which has not undergone any revision since 1994 [18, 19]. This ordinance regulates the amount of theoretical and practical training as well as the state examinations. Details on the transfer of evidence-based content or the knowledge and application of clinical guidelines are not addressed [18, 19]. While other countries in Europe initiated bachelor’s degree programmes as entry levels for the physiotherapy profession around 70 years ago, the first such programmes were not offered in Germany until 2001 following the signing of the Bologna Agreement in 1999. Today, primary qualifying study courses are still subject to evaluation until 2024 [20]. In 2019, there were approximately 203,000 physical therapists in Germany providing care to patients, of whom only 2000 had an academic degree [17]. Also, unlike the case in other countries, physical therapy in Germany is considered an assistant profession, while the practice of physical therapy for the purposes of stroke rehabilitation, regardless of whether physiotherapists have a university education or not, requires a doctor’s prescription. The access to, level and content of physical practice therefore depends a great deal on the priorities of a physician.

Many guidelines are available to inform stroke rehabilitation and enable evidence-based practice both in Germany and the wider world [21–23]. Clinical guidelines are defined as” statements that include recommendations intended to optimise patient care” [24]. These statements are based on systematic literature reviews as well as the weighed advantages and disadvantages of medical procedures [24]. Even though physical therapy compliant with guidelines enhances walking speed and walking endurance after stroke, guidelines appear to be implemented inconsistently the world over [25–29].

The ‘Rehabilitation of Mobility after Stroke (ReMoS)’ guideline was developed by neurologists and physical therapists of the German Society of Neurorehabilitation (Deutsche Gesellschaft für Neurorehabilitation - DGNR) and published by the ‘Working Group of Scientific Medical Societies’ (Arbeitsgemeinschaft Wissenschaftlicher Medizinischer Fachgesellschaften - AWMF) [22, 30]. The authors of the ReMoS guideline make over 250 evidence-based recommendations within five goal-directed domains: ‘Achieving the ability to walk for non-ambulant patients, ‘Improving the ability to walk in (partially) ambulant patients’, ‘Improving of walking speed’, ‘Improving of walking distance’ and ‘Improving of balance and reducing the risk of falls’ distinguished by the subacute and chronic phase after stroke [31]. For this guideline, subacute stroke was defined as the six-month period after the event. The development of the guideline followed a systematic literature research and classification of the evidence according to the ‘Grades of Recommendation, Assessment, Development and Evaluation (GRADE)’ approach. In consideration of adverse events as well as clinical applicability, a recommendation was derived (A = shall be used, B = should be used, 0 = can be used or no recommendation) (see Additional file 1) [32]. The recommendations assigned to the five domains mentioned are not explicitly directed towards specific health care professions. The recommendations rated A or B for the subacute phase after stroke, however, are particularly strongly related to the physiotherapy’s field of action. Recommendations among those five domains for the subacute phase after stroke include, e.g., ‘intensive walking training using electromechanical assistance’, ‘task-specific endurance training’, ‘home exercise programs’, ‘walking training with reflex-based functional electrical stimulation’ and ‘motor relearning programs’ and address the field of action of the physical therapy.

Passive and unstructured strategies were employed to disseminate and publicise this guideline. It is available as a full version of 144 pages as well as a short version of 6 pages both in print and in a free downloadable digital format [30, 32]. After the survey was conducted, a website optimised for mobile devices was launched by the DGNR and may have feasibly contributed towards the awareness and usability of the ReMoS guideline [33].

At the time the project was developed, the ReMoS guideline provided the highest evidence-level relating to the rehabilitation of post-stroke mobility in Germany [22, 34]. Since the degree of awareness, the level of its implementation, and related implementation factors are all unknown, our objective was:

1. to describe self-reported adherence of physical therapists working in Germany to ReMoS guideline recommendations for the subacute phase after stroke, and
2. to explore the barriers against and facilitators of guideline usage.

## Methods

The reporting of the survey methods and results follows the ‘Strengthening the Reporting of Observational Studies in Epidemiology (STROBE)’ statement and the ‘Checklist for Reporting Results of Internet E-Surveys (CHERRIES)’ [35, 36]. Ethical approval was obtained from the ethics committee of the Brandenburg University of Technology Cottbus-Senftenberg (BTU C-S), Germany (EK2019–5), and the study was retrospectively registered to the German Clinical Trials Register (DRKS00026681).

### Study scope

The study was designed to evaluate and describe current physical therapy practice in German stroke rehabilitation with regard to the ReMoS guideline and represents a component of the pre-implementation planning [37]. Knowledge of local determining factors will inform the design of implementation strategies [38–40].

### Study design

This descriptive cross-sectional study was conducted as a nationwide open online survey addressed at physical therapists working in Germany.

### Questionnaire development

A self-administered web-based questionnaire was developed based on the ReMoS guideline and the ‘Barriers and Facilitators Assessment Instrument (BFAI)’ [32, 41].

A preliminary questionnaire of three sections was drafted, aligned to the recommendations by Dillman et al. [42], and was critically revised by the study team as well as the ReMoS working group of the DGNR. Pre-testing took place with seven physical therapists using the cognitive technique referred to as ‘post-interview probing’ [43], whereupon additional freely formulated response options and further perceived barriers regarding the use of ReMoS recommendations were incorporated. The final online survey was prepared using the browser-based software ‘LimeSurvey’ and consisted of 12 pages (including a welcome page and a closing page, information about the study project and privacy protection, and a page for declaring informed consent) with 89 open and closed questions in a fixed sequence (see Additional file 2 for the survey in German language and Additional file 3 for the translated English version of the questionnaire). Textual information about the purpose, extent, anonymous data storage, and analysis was provided on the welcome screen. The survey questions were structured into three sections. As with earlier studies, participants were asked to rate the implementation of the ReMoS guideline recommendations regarding physical therapy in the subacute phase after stroke on an ordinal scale (always, often, sometimes and never) in the first section [44, 45]. In the second section, the validated BFAI by Peters et al. [41] was translated pragmatically to explore the determining factors of ReMoS guideline usage. The BFAI was developed to identify facilitators for and barriers against the implementation of preventive care, innovations, or guidelines. The original instrument was based on a literature review and an expert panel consensus procedure [41]. In the third and final section, characteristics of participants and their occupational circumstances (e.g. age, gender, years of job experience and occupational location) were collected. The survey extent varied as an adaptive answering procedure was applied to limit the burdens on the study participants. Participants who stated that they were not aware of the ReMoS guideline were forwarded to the final section on demographic characteristics, and in so doing were not asked about barriers and facilitators. Based on the preliminary tests, it was assumed that the questionnaire would take 20 minutes to complete. Apart from the approval of the eligibility criteria, participants always had the opportunity to refuse to answer and were able to navigate within the screens. While the survey was being completed, the level of completeness was presented visually and in numerical form. IP-addresses were not logged and no cookies were used, allowing multiple accesses to the survey via the same internet connection (e.g. from several physical therapists using the same computer). Data was stored and analysed on password-protected servers of the BTU Cottbus-Senftenberg. Intermediate storage on an access-restricted ‘LimeSurvey’ database was immediately deleted once recruitment was completed. Where participants had provided their email addresses voluntarily, these were stored separately from the survey data. The questionnaire was only available in the German language. Due to the scope of the study, an evaluation of the questionnaire validity and reliability was not conducted.

### Participants

Physical therapists were invited to participate voluntarily with no provision of incentives. For accessing the survey, all participants approved the following self-reported eligibility criteria, without any further checking.

1. qualification as a physical therapist,
2. currently working in in outpatient or inpatient stroke rehabilitation,
3. treating people with a subacute stroke

There is no register for physical therapists in Germany. However, it is estimated that about 200,000 people work in this profession [17]. As patients after stroke are treated by physical therapists with a range of treatments and in different health care settings, the precise number of eligible physical therapists is unknown. Since hypotheses were not tested, sample size calculations were not conducted. To achieve representativeness, we aimed to recruit as many eligible physical therapists as possible.

### Survey implementation

Data were collected between October and December 2019. The link to the browser-based online survey was distributed via 1) e-mail to 248 in-patient rehabilitation clinics specialised in neurorehabilitation in Germany, 2) professional societies (Deutscher Verband fuer Physiotherapie (ZVK) e.V., & Verband Physikalische Therapie – Vereinigung fuer die physiotherapeutischen Berufe (VPT) e.V.), 3), postings on the most accessed online physical therapy forum in Germany (www.physio.de), 4) social media accounts of the German Society for Physiotherapy Science (DGPTW e.V.) 5) cooperating partners of the BTU Cottbus-Senftenberg, and directly to professional personal contacts. Any further dissemination beyond these means was also encouraged (e.g. ‘snowball-sampling’). The survey was accessible for 6 weeks. Within that time, two acknowledgements and reminders were sent via the same channels after 2 and 4 weeks. Before access was granted to the survey, participants had to provide their written informed consent.

### Data analysis

Data from the online survey were exported to Microsoft Excel and checked for plausibility. Data analysis was conducted with R and R Studio version 3.4.0. All the questionnaires which were answered at least in part (confirmation of the eligibility criteria and response to at least one item) were analysed. We did not correct for any missing data. Approval for further contact and feedback on the questionnaire were not analysed. Participation and completion rates were calculated on the basis of the number of survey accesses, agreements to participate, and the number of participants submitting the final survey page.

Demographic characteristics were analysed based on the item response and data level. Categorical variables are reported as absolute and relative frequencies, while for continuous variables the means and standard deviations are presented.

Guideline adherence can be defined as “the degree of conformity between the knowledge, cognition and/or action of an agent” with guideline recommendations [46]. As was the case in earlier studies, the evaluation of guideline adherence was based on the actions of physical therapists with regard to individual guideline recommendations [27, 47]. Frequencies and percentages were calculated per guideline recommendation, within each domain, and for overall guideline adherence. To evaluate the levels of guideline adherence per ReMoS guideline recommendation, results were dichotomized as “always” and “often” being guideline adherent and “sometimes” and “never” being not guideline adherent. Guideline adherent physical therapy practice was defined as 80% of the responders reporting frequent implementation of guideline recommendations. This level was characterised as being ‘excellent’ by Donohue et al., 2014 [47].

The first author of this paper coded the meaning of the freely written text entries regarding 1) the physiotherapeutic interventions designed to achieve post-stroke mobility-related rehabilitation goals, and 2) the access options to the guideline. The codings were then analysed for their frequencies within the guideline domains and reported descriptively.

Responses to the BFAI questionnaire were analysed and presented for each item as absolute and relative frequencies based on the item response rate. To explore barriers and facilitators to ReMoS guideline use, responses of “fully agree” and “agree” as well as “disagree” und “fully disagree” were merged. Responses to the option “ do not agree nor disagree” were not included within this dichotomization, consistent with the recommendations of the questionnaire developers [41].

Sample characteristics were described descriptively according to the data scale level, where for each item an appropriate point measure as well as a scattering measure are given.

## Results

The survey was accessed 323 times. Eligibility criteria were fulfilled by 242 persons, resulting in a participation rate of 74%. The completion rate was 40%, as only 97 participants finished the last survey page. Because of the open sampling design, an exact response rate could not be calculated. Data from 170 questionnaires were eligible for analysis. One questionnaire was removed because of its implausibility.

### Participants’ characteristics

Participants were on average 42 years of age (standard deviation 11.4) years old, mostly female (*n* = 68, 69.4%), employed in a rehabilitation centre (50, 51.5%) and had no academic degree (*n* = 57, 60.1%)). Table 1 provides detailed information on participants´ characteristics.

**Table 1:** Characteristics of the participants

Characteristics; Item response	Mean (SD)	Number of participants (%)
Age in years; *n* = 97	41.6 (11.4)	
Job experience in years; *n* = 96	15.5 (15.7)	
Weekly working time in hours; *n* = 97	34.6 (11.1)	
Gender; *n* = 98		
Female		68 (69.4)
Male		30 (30.1)
Working environment; *n* = 94		
Large City (> 100,000 inhabitants)		23 (24.5)
Town (5000–20,000 inhabitants)		20 (21.3)
Suburban (20,000–100,000 inhabitants)		37 (39.4)
Rural (< 5000 inhabitants)		14 (14.9)
Working place; *n* = 97		
Rehabilitation centre		50 (51.5)
Private physiotherapy practice		32 (33)
Others		15 (15.5)
Employment; *n* = 96		
Employees		76 (79.2)
Self-employed		19 (19.8)
Freelancer		1 (1.4)
Highest degree; *n* = 94		
No academic degree		57 (60.1)
Bachelor		15 (16)
Master		9 (9.6)
Diploma		7 (7.4)
Doctorate/ PhD		6 (6.4)
Management position; *n* = 93		50 (53.8)
Multi-professional team; *n* = 93		78 (83.9)
Number of patients with stroke on an average working day; *n* = 95		
< 5		51 (53.7)
6–10		35 (36.8)
> 10		9 (9.5)
Time per treatment session (minutes); *n* = 97		
< 20		2 (2.1)
21–30		51 (52.6)
31–45		30 (30.9)
> 45		14 (14.4)

The ReMoS guideline was unknown to 52.9% (*n* = 55/104) of the responders. Twenty-five persons indicated how they became aware of the guideline and reported academic education (*n* = 7), online research (*n* = 6), their workspace (*n* = 5) and others (*n* = 14) as their main familiarising sources.

### Guideline adherence

Overall guideline adherence was 34.8%. The highest levels of adherence were reported within the domain ‘Improving the ability to walk in (partially) ambulant patients’, and the lowest levels were reported within the domain ‘Achieving the ability to walk for non-ambulant patients’.

Guideline adherent physical therapy as defined in this study was identified for only intensive walking training for ‘Improving of balance and reducing the risk of falls’ (84.3%, *n* = 86/102)). The lowest levels of adherence were reported for walking training with functional electric stimulation within the domains ‘Improving of walking distance’ (1.9%, *n* = 2/108), ‘Improving the ability to walk in (partially) ambulant patients’ (5.5%, *n* = 8/145) and a combination of end-effector-based devices with functional electric stimulation for ‘Improving of walking speed’ (2.4%, *n* = 3/124).

Intensive walking training was reported to be used frequently, but not in combination with technical support or functional electrical stimulation as recommended in the ReMoS guideline. Most of the study participants reported using walking aids for ‘Improving the ability to walk in (partially) ambulant patients’ as well as acoustic feedback while walking. Further results are presented in Table 2.

**Table 2:** Self-reported adherence to overall ReMoS guideline, guideline domains and guideline recommendations

Level of recom-mendation	ReMoS recommendation, Item response	never n (%)	sometimes n (%)	often n (%)	always n (%)	Level of guideline adherence (%)
Overall ReMoS guideline adherence						34.8
Achieving ability to walk in non-ambulant patients						18.9
B	Intensive walking training; *n* = 168	13 (7.7)	31 (18.5)	83 (49.4)	41 (24.4)	73.8
B	Intensive walking training using end-effector-based device; *n* = 161	82 (50.9)	39 (24.2)	27 (16.8)	13 (8.1)	24.8
0	Intensive walking training using treadmill or exoskeleton; *n* = 161	74 (46)	57 (35.4)	24 (14.9)	6 (3.7)	18.6
0	Functional electric stimulation in supine position; *n* = 161	116 (72)	33 (20.5)	10 (6.2)	2 (1.2)	7.5
Improving ability to walk in (partially) ambulant patients						42.3
B	Intensive walking training (conventionally); *n* = 146	8 (5.5)	25 (17.1)	61 (41.8)	52 (35.6)	77.4
B	Intensive progressive walking training using treadmill; *n* = 144	45 (31.2)	56 (38.9)	31 (21.5)	12 (8.3)	29.9
0	Task-specific training with motor imagery; *n* = 146	18 (12.3)	65 (44.5)	42 (28.8)	21 (14.4)	43.2
0	Walking aids; *n* = 149	1 (0.7)	36 (24.2)	85 (57.0)	27 (18.1)	75.2
0	Walking training with functional electrical stimulation; *n* = 145	123 (84.8)	14 (9.7)	6 (4.1)	2 (1.4)	5.5
Improving walking speed						34.6
A	Task-specific endurance training using treadmill; *n* = 128	41 (32.0)	45 (35.2)	31 (24.2)	11 (8.6)	32.8
A	Task-specific endurance training using progressive circuit training; *n* = 124	45 (36.3)	42 (33.9)	26 (21)	11 (8.9)	29.8
B	Intensive walking training without treadmill; *n* = 130	6 (4.6)	29 (22.3)	70 (53.8)	25 (19.2)	73.1
B	Intensive walking training using treadmill; *n* = 125	42 (33.6)	44 (35.2)	35 (28)	4 (3.2)	31.2
B	Home exercise program; *n* = 123	19 (15.4)	26 (21.1)	55 (44.7)	23 (18.7)	63.4
B	Walking training with functional electrical stimulation; *n* = 121	99 (81.8)	15 (12.4)	5 (4.1)	2 (1.7)	5.8
B	Additional training for lower extremity functions; *n* = 128	6 (4.7)	40 (31.3)	59 (46.1)	23 (18)	64.1
0	Intensive progressive task-specific training; *n* = 125	2 (1.6)	33 (26.4)	61 (48.8)	29 (23.2)	72
0	Task-specific training with motor imagery; *n* = 129	25 (19.4)	56 (43.4)	35 (27.1)	13 (10.1)	37.2
0	Walking training using end-effector devices; *n* = 96	59 (96.4)	20 (22)	16 (13.8)	1 (0.8)	17.7
0	Muscular endurance training; *n* = 130	0 (0)	46 (35.4)	60 (46.2)	24 (18.5)	64.6
0	Isokinetic strength training; *n* = 128	48 (37.5)	51 (39.8)	25 (19.5)	4 (3.1)	22.7
0	Walking training with acoustic stimulation; *n* = 129	33 (25.6)	75 (58.1)	17 (13.2)	4 (3.1)	16.3
0	Acoustic feedback while walking; *n* = 129	43 (33.3)	49 (38)	27 (20.9)	10 (7.8)	28.7
0	Feedback/ Reinforcement; *n* = 124	57 (46)	46 (37.1)	19 (15.3)	2 (1.6)	16.9
0	Combination of end-effector-based devices with functional electric stimulation; *n* = 124	107 (86.3)	14 (11.3)	3 (2.4)	0 (0)	2.4
0	Early use of ankle-foot-orthosis; *n* = 130	11 (8.5)	77 (59. 2)	39 (30)	3 (2.3)	32.3
0	Early use of orthopedic shoes; *n* = 127	39 (30.7)	65 (51.2)	21 (15.7)	3 (2.4)	18.9
0	Arm slings; *n* = 125	22 (17.6)	65 (52	30 (24)	8 (6.4)	30.4
Improving walking distance						37.2
A	Task-specific endurance training; *n* = 118	1 (0.8)	38 (32.2)	58 (49.2)	21 (17.8)	67
B	Home exercise program; *n* = 115	14 (12.2)	34 (29.6)	47 (40.9)	20 (17.4)	58.3
B	Intensive walking training using treadmill; *n* = 113	54 (47.8)	37 (32.7)	14 (12.4)	8 (7.1)	19.5
0	Task-specific training with motor imagery; *n* = 116	33 (28.4)	50 (43.1)	27 (23.3)	6 (5.2)	28.5
0	Walking training with end-effector-based device; *n* = 109	74 (67.9)	21 (19.3)	13 (11.2)	1 (0.9)	12.8
0	Muscular endurance training; *n* = 116	2 (1.7)	44 (37.9)	51 (44)	19 (16.4)	60.3
0	Walking training with functional electrical stimulation; *n* = 108	92 (85.2)	14 (13)	1 (0.9)	1 (0.9)	1.9
0	High frequent physical therapy at home; *n* = 98	57 (58.1)	17 (17.3)	15 (15.3)	9 (9.3)	24.6
0	Additional training for lower extremity functions; *n* = 115	9 (7.8)	51 (44.3)	42 (36.5)	13 (11.3)	47.8
0	Additional muscular endurance training; *n* = 112	12 (10.7)	49 (43.7)	38 (33.9)	13 (11.6)	45.5
Improving balance and reducing risk of falls						39.1
B	Intensive walking training without treadmill; *n* = 102	3 (2.9)	13 (12.7)	53 (52)	33 (32.4)	84.3
B	Intensive walking training using treadmill; *n* = 100	34 (34)	37 (37)	27 (27)	2 (2)	29
B	Home exercise program; *n* = 98	14 (14.3)	34 (34.7)	34 (34.7)	16 (16.3)	51
B	Motor relearning program; *n* = 97	18 (18.6)	27 (27.8)	36 (37.1)	16 (16.5)	53.6
0	Walking training using end-effector-based device, exoskeleton or treadmill; *n* = 96	59 (61.5)	20 (20.8)	16 (16.7)	1 (1)	17.7
0	Muscular endurance training; *n* = 99	18 (18.2)	33 (33.3)	35 (35.4)	13 (13.1)	48.5
0	Trunk control training on unstable surface; *n* = 100	4 (4)	43 (43)	34 (34)	19 (19)	53
0	Acoustic feedback while walking; *n* = 98	4 (4.1)	23 (23.5)	47 (48)	24 (24.5)	72.5
0	Early use of orthopedic shoes; *n* = 96	63 (65.6)	22 (22.9)	7 (7.3)	4 (4.2)	11.5
0	Additional ergometer training; *n* = 95	33 (34.7)	40 (42.1)	15 (15.8)	7 (7.4)	23.2
0	Additional training on a biofeedback platform; *n* = 98	35 (35.7)	46 (46.9)	15 (15.3)	2 (2)	17.4

*Abbreviation*: *ReMoS* Rehabilitation of Mobility after Stroke

0 = “can be applied”, A = “shall be applied”, B = “should be applied”

Sixty-seven participants provided more detailed insights into their daily practice by responding to open-ended questions on therapeutic goal-directed interventions. Interventions based on traditional rehabilitation concepts (e.g. Bobath, Vojta, Proprioceptive Neuromuscular Facilitation) were reported to be used often for ‘Achieving the ability to walk in non-ambulant patients’. Exercises on unstable or varying surfaces were reported to be used for ‘Improving of balance and reducing the risk of falls’ (see Additional file 3).

### Barriers and facilitators of guideline use

Table 3 displays the reported barriers and facilitators of ReMoS guideline usage. Only participants who reported as being aware of the guideline were directed to this section of the survey. In terms of professional related factors, the majority of the respondents disagreed with the statements on lacking knowledge (*n* = 32, 84.2%), the ReMoS-guideline is not fitting into daily routines (*n* = 30, 81.1%), having problems with changing routines (*n* = 30, 71.5%) or a general reluctance to work according to protocols (*n* = 30, 71.4%), which may facilitate the ReMoS guideline use. Further, participants acknowledged the guideline being a good starting point for their self-study (*n* = 23, 57.5%), leaving adequate space to draw their own conclusions (*n* = 19, 46.4%) and to weigh the wishes of the patients (*n* = 18, 43.9%). Twenty respondents (60.6%) agreed on a need for financial compensation as barrier when having to work according to the ReMoS guideline.

**Table 3:** Perceived barriers and facilitators of ReMoS guideline use

Item, Item response	Fully disagree; n (%)	Disagree; n (%)	Do not agree nor disagree; n (%)	Agree; n (%)	Fully agree; n (%)
The ReMoS guideline leaves enough room for me to make my own conclusions; *n* = 41	1 (2.4)	2 (4.9)	19 (46.3)	15 (36.6)	4 (9.8)
The ReMoS guideline leaves enough room to weigh the wishes of the patient; *n* = 41	1 (2.4)	7 (17.1)	15 (36.6)	16 (39)	2 (4.9)
The ReMoS guideline is a good starting point for my self-study; *n* = 40	3 (7.5)	5 (12.5)	9 (22.5)	12 (30)	11 (27.5)
I did not thoroughly read nor remember the ReMoS guideline; *n* = 42	10 (23.8)	13 (31)	14 (33.3)	2 (4.8)	3 (7.1)
I wish to know more about the ReMoS guideline before I decide to use it^a^; *n* = 38	18 (47.4)	14 (36.8)	4 (10.5)	0 (0)	2 (5.3)
I have problems changing my old routines; *n* = 42	13 (31)	17 (40.5)	11 (26.2)	1 (2.4)	0 (0)
I think parts of the ReMoS guideline are incorrect; *n* = 36	7 (19.4)	9 (25)	13 (36.1)	5 (13.9)	2 (5.6)
I have a general resistance to working according to protocols; *n* = 42	16 (38.1)	14 (33.3)	7 (16.7)	2 (4.8)	3 (7.1)
Fellow physical therapists do not cooperate in using the ReMoS guideline^a^; *n* = 36	3 (8.3)	8 (22.2)	13 (36.1)	9 (25)	3 (8.3)
Neurologists and other physicians do not cooperate in using the ReMoS guideline^a^; *n* = 32	4 (12.5)	6 (18.8)	12 (37.5)	9 (28.1)	1 (3.1)
Managers/directors do not cooperate in using ReMoS guideline^a^; *n* = 31	10 (32.3)	5 (16.1)	8 (25.8)	5 (16.1)	3 (9.7)
Patients do not cooperate in using the ReMoS guideline^a^; *n* = 35	3 (8.6)	12 (34.3)	14 (40)	5 (14.3)	1 (2.9)
Working according to the ReMoS guideline is too time consuming; *n* = 38	3 (7.9)	11 (28.9)	11 (28.9)	10 (26.3)	3 (7.9)
The ReMoS guideline does not fit into my ways of working in daily practice^a^; *n* = 37	20 (54,1)	10 (27)	6 (16.2)	1 (2.7)	0 (0)
Working according to the ReMoS guideline requires financial compensation; *n* = 33	5 (15.2)	5 (15.2)	3 (9.1)	10 (30.3)	10 (30.3)
The lay-out of the ReMoS guideline makes it handy for use; *n* = 29	1 (2.6)	4 (10.3)	12 (30.8)	12 (30.8)	0 (0)

*Abbreviation*: *ReMoS* Rehabilitation of Mobility after Stroke

^a^ = The original statements were rephrased for the content and purpose of the survey

Of the additional barriers, the availability of end-effector or exoskeleton-based devices and treadmills were relevant factors to 56.8–84.1% of the responders. Only 31.8% (*n* = 14) and 15.6% (*n* = 7) of the responders, respectively, felt that they required knowledge of specific recommendations or that they were unaware of the link between the guideline and the phases of neurorehabilitation in Germany. Involvement of other professions was reported to be relevant by 46.7% (*n* = 21) of the responders.

## Discussion

The survey explored self-reported adherence to ReMoS guideline recommendations as well as influencing factors. Many physical therapists (52.9%, *n* = 55/104) reported that they were aware of the guideline. Overall guideline adherence was 34.8% and varied according to the individual recommendation. Only one ReMoS guideline recommendation was reported to be adhered to by more than 80% of physical therapists, highlighting the deficits in guideline adherence. Recommendations including technical support were reported to be used less frequently. Barriers for guideline use were usually denied. Participants predominantly agreed on positive guideline-related factors, such as the ReMoS guideline being a good starting point for their self-study, leaving adequate space for making their own conclusions and respecting patient preferences.

### Participant characteristics

Our sample is comparable to the general population of physical therapists in Germany in terms of age, sex distribution and working setting, but substantially over represents those physical therapists with an academic qualification (39.4% in our sample compared to approximately 3% nationally) [17, 48].

Although the ReMoS guideline was published 4 years before the survey, many physical therapists were still unaware of it. Since no systematic or theory-based approach was used to disseminate or implement the ReMoS guideline, physical therapists may not have been reached via typical passive dissemination routes [49]. Also, structural limitations may also have been an underlying factor, as Bahns et al. [44] also reported with the lack of awareness of a guideline for non-specific low back pain among German physical therapists. Most physical therapists in Germany graduate from vocational schools, where the training content is based on the 1994 Training and Examination Ordinance for Physiotherapists (PhysTh-AprV). This may result in a lack of conveyance of knowledge about guidelines as well as a limited acquisition of competence for evidence-based practice. Based on current German law, patients with statutory health insurance have a right to care that is consistent with the current state of scientific knowledge (Social Code Book Five - Statutory Health Insurance § 135a). An amendment of the professional laws, which is expected in 2024, might make the full or partial academic training of physiotherapists in Germany permanent. Until then, however, owners of physiotherapy practices, managers of therapeutic facilities, and the physiotherapists themselves should prioritise the financing of evidence-based, practice-oriented, advanced training.

### Guideline adherence

As guidelines represent only one way by which evidence-based knowledge is transferred into practice, adherence to the ReMoS guideline was calculated regardless of whether physical therapists were aware of the guideline or not. In the light of the self-selected participation and the self-reported information, frequencies of adherence for some recommendations can be considered as good to excellent [25, 47]. Because our sample conveniently over-represented professionals with an academic degree, our results are also likely to overestimate the true level of guideline adherence amongst all physical therapists [44].

Similar to our findings and consistent with Donnellan et al. [50], adherence to guidelines seems to vary across recommendations amongst European nurses, speech therapists, and physical therapists involved in acute stroke care and stroke rehabilitation [28, 47, 51–53]. The varying adherence to the recommendations in our survey might in part be explained by the underrepresentation of other health professions involved in post-stroke mobility rehabilitation. For example, sports therapists can also implement recommendations such as gait training with technical support, and functional electrical stimulation can be performed by masseurs and balneotherapists in Germany. The ReMoS guideline itself does not distinguish responsibilities.

Although Germany differs significantly from other countries regarding the education level for physiotherapists and autonomy in professional practice, this alone cannot explain the restricted adherence to the guidelines. Studies in Europe and worldwide point to the limitations of guideline-based physiotherapy in post-stroke rehabilitation [29, 54, 55].

However, the heterogeneous use of the concept ‘guideline adherence’ and the insufficiently standardised evaluation and operationalisation of the concept should be looked at critically. The resulting restricted comparability of study results has been criticised by experts within the context of physiotherapy research [56, 57]. Varying methods and benchmarks for evaluating guideline adherence can also be seen specifically in the context of stroke rehabilitation. This applies to physiotherapy in stroke research, where as an alternative to our and similar studies using self-reported data, retrospective [27, 28, 58] and prospective audits [25, 29] are used to describe practices related to guideline usage.

### Barriers and facilitators

Barriers to guideline adherence and evidence-based physiotherapy have been evaluated elsewhere. These include factors related to health care professional characteristics, the context, and the guideline itself [50, 59–61].

In line with previous studies, we used the BFAI to quantitatively evaluate the influencing factors of guideline use in stroke rehabilitation [51, 62]. In our sample, many factors were found to be similar to those identified by Otterman et al. [51] and van Peppen et al. [62],e.g., physical therapists’ sense of having enough knowledge about the guideline, that the guideline leaves enough room to respect patients’ preferences and allows physical therapists to draw their own conclusions, as well as the agreement regarding financial compensation. In contrast to the previously mentioned studies, participants in our study did not report inadequate time or problems with changing routines.

As reported by Ajimsha et al., the lack of availability of therapeutic equipment can also lead to a limited application of ReMoS recommendations [27]. The authors there report that the lack of therapeutic devices explains adherence rates of 0% regarding electromyographic biofeedback, robotic, and virtual reality training. Less equipment-intensive interventions, such as circuit class training for walking (95%), was on the other hand used more often [27]. This emphasises the value of the current literature where barriers and facilitators have been assessed not in relation to an entire guideline, but rather in relation to individual recommendations [63–65]. As the authors of the ReMoS guideline recommend several resourceful interventions targeting different rehabilitation goals, a more refined exploration at the level of individual recommendations might also prove useful.

Knowledge about factors influencing guideline usage in stroke rehabilitation usually emerges from multidisciplinary qualitative or mixed-methods approaches, where aspects such as ‘practical familiarity with the recommended treatments’, environmental factors (e.g. time and resources), guideline characteristics (e.g. ‘limited applicability of recommendations’), or organisational or personal factors with respect to patients, relatives and therapists [59, 66–73] are reported on. Building upon our results, additional approaches such as focus groups [72, 73], semi-structured interviews [67, 69], or both [68], should serve to broaden or further specify aspects of the ReMoS guideline.

### Limitations

Using online-survey methods for this cross-sectional design is afflicted with several limitations.

Sampling was specifically directed towards physiotherapists who work in outpatient and inpatient rehabilitation settings with patients in a subacute phase following a stroke. Therefore, the results of the survey do not represent the entire population of German physiotherapists. Although we attempted to distribute the survey to a large number of physical therapists using an open sampling approach, the number of study participants (*n* = 170) was small compared to the total number of approximately 200,000 physical therapists working in Germany [17, 74]. However, surveys conducted in Germany also recruited comparatively small samples related to the total number of physical therapists in Germany [44, 75]. As there is no register for physiotherapists in Germany, it is unclear how large the proportion of those working with neurological patients is. In addition, only the perspective of physiotherapists was considered and other professional groups that might be involved in implementing the ReMoS guideline were omitted.

Furthermore, it is unclear how valid the results regarding barriers and facilitators are, since the translation, cultural adaption and validation of the BFAI are all still pending. Since only those physical therapists who were unaware of the ReMoS guideline were directed directly to the final section on participant characteristics, their perception of the barriers against and facilitators for using the ReMoS guideline remains unknown.

By using an open-access online survey and distributing it through multiple channels, as well as sending multiple reminders, we attempted to avoid any sampling bias. At the same time, multiple responding could not be avoided because of the design of the open survey as being without restricted access. Apart from employing a ‘snowball-sampling’ paradigm, additional ways of collecting survey data, such as via postal letters or via phone calls, might have further increased the participation rate.

The survey aimed to capture several aspects of physiotherapy practice as related to the ReMoS guideline. Despite the adaptive answering procedure, the number of questions and the predominantly closed question format may have negatively influenced the flow of participants and the overall response rate.

Because of the self-selected participation and the self-reported data, our results will not provide the whole picture of stroke rehabilitation in Germany. Acquisition bias and social desirability bias due to self-reported answering could well be ameliorated by chart audits or clinical observations [76].

### Implications

Future studies might employ qualitative approaches to acquire a comprehensive understanding of determining factors, and might also enable the development of multifaceted interventions to implement the ReMoS guideline in a targeted manner. A consistent definition of guideline adherence by health care professionals will lead to an enhanced comparability of the results.

As the survey was not designed to investigate adherence to ReMoS recommendations during the chronic phase after stroke, and as it did not consider the multidisciplinarity of stroke rehabilitation, further investigations across the stroke rehabilitation continuum in Germany are needed. Given the paucity of implementation studies regarding stroke rehabilitation and the fact that the most effective implementation interventions have yet to be discovered, implementation researchers should investigate the feasibility, effectiveness and sustainability of implementation interventions under the specific conditions of the German health care system [77, 78].

In the German context, a secure legal legitimisation of academic physiotherapy education as well as a targeted dissemination of guidelines to users should increase the awareness of these guidelines [79]. Politicians and managers of rehabilitation facilities need to be aware of existing problems and invest in the rehabilitation infrastructure as well as health care professionals to ensure a guideline adherent and thus evidence-based rehabilitation.

## Conclusions

Among German physical therapists, the ReMoS guideline is largely unknown. Self-reported guideline adherence, as defined here, was only seen for the recommendation on intensive walking training to improve balance and reducing the risk of falls. Recommendations about interventions that require technical equipment in particular are rarely implemented according to the physiotherapists. A more in-depth investigation of the determining factors is needed, as restricted knowledge, problems with changing routines, or a general reluctance to work according to protocols were usually denied. Better funding of guideline-based physiotherapy could be a priority at the level of the health system and management responsibility in both rehabilitation clinics and physiotherapy practices.

## Supplementary Information

**Additional file 1.** Process of guideline development according to Dohle et al., 2016 [32].

**Additional file 2. ** Original German version of the questionnaire.

**Additional file 3. ** Translated English version of the questionnaire.

**Additional file 4.** Responses on open-ended questions according therapy-goal directed interventions within ReMoS guideline domains.

## Data Availability

The data sets generated and/or analysed during the current study are not publicly available due to an explicit exclusion in the consent form, but can be requested from the corresponding author upon reasonable request.

## Abbreviations

BFAI: Barriers and Facilitators Assessment Instrument
BTU C-S: Brandenburg University of Technology Cottbus-Senftenberg
CHERRIES: Checklist for Reporting Results of Internet E-Surveys
DGNR: German Society of Neurorehabilitation
DGPTW: German Society for Physiotherapy Science
e.V: Eingetragener Verein
GRADE: Grades of Recommendation, Assessment, Development and Evaluation
PhysTh-AprV: Training and Examination Ordinance for Physiotherapists
ReMoS: Rehabilitation of Mobility after Stroke
SD: Standard deviation
STROBE: Strengthening the Reporting of Observational Studies in Epidemiology Statement
VPT: Verband Physikalische Therapie – Vereinigung fuer die physiotherapeutischen Berufe
ZVK: Deutscher Verband fuer Physiotherapie

## References

[1] Collaborators GS (2021). Global, regional, and national burden of stroke and its risk factors, 1990-2019: a systematic analysis for the global burden of disease study 2019. Lancet Neurol.

[2] Wafa HA, Wolfe CDA, Emmett E, Roth GA, Johnson CO, Wang Y (2020). Burden of stroke in Europe: thirty-year projections of incidence, prevalence, deaths, and disability-adjusted life years. Stroke..

[3] Robert-Koch-Institut. Gesundheit in Deutschland. Gesundheitsberichterstattung des Bundes. Gemeinsam getragen von RKI und Destatis. Berlin: RKI; 2015. 10.17886/rkipubl-2015-003-2.

[4] Lo J, Chan L, Flynn S (2021). A systematic review of the incidence, prevalence, costs, and activity and work limitations of amputation, osteoarthritis, rheumatoid arthritis, Back pain, multiple sclerosis, spinal cord injury, stroke, and traumatic brain injury in the United States: a 2019 update. Arch Phys Med Rehabil.

[5] Pollock A, St George B, Fenton M, Firkins L (2014). Top 10 research priorities relating to life after stroke–consensus from stroke survivors, caregivers, and health professionals. Int J Stroke.

[6] Rudberg AS, Berge E, Laska AC, Jutterström S, Näsman P, Sunnerhagen KS (2021). Stroke survivors' priorities for research related to life after stroke. Top Stroke Rehabil.

[7] Louie DR, Simpson LA, Mortenson WB, Field TS, Yao J, Eng JJ. Prevalence of walking limitation after acute stroke and its impact on discharge to home. Phys Ther. 2022;102(1):pzab246. 10.1093/ptj/pzab246. 10.1093/ptj/pzab246 PMC8787995 34718796

[8] Cohen JW, Ivanova TD, Brouwer B, Miller KJ, Bryant D, Garland SJ (2018). Do performance measures of strength, balance, and mobility predict quality of life and community reintegration after stroke?. Arch Phys Med Rehabil.

[9] Kwakkel G, Kollen BJ, van der Grond J, Prevo AJ (2003). Probability of regaining dexterity in the flaccid upper limb: impact of severity of paresis and time since onset in acute stroke. Stroke..

[10] Harris JE, Eng JJ (2004). Goal priorities identified through client-Centred measurement in individuals with chronic stroke. Physiother Can.

[11] Shum ST, Chiu JK, Tsang CP, Wong CH, Tsang RC, Ma SL (2014). Predicting walking function of patients one month poststroke using modified Rivermead mobility index on admission. J Stroke Cerebrovasc Dis.

[12] Lord SE, McPherson K, McNaughton HK, Rochester L, Weatherall M (2004). Community ambulation after stroke: how important and obtainable is it and what measures appear predictive?. Arch Phys Med Rehabil.

[13] Pollock A, Baer G, Campbell P, Choo PL, Forster A, Morris J, et al. Physical rehabilitation approaches for the recovery of function and mobility following stroke. Cochrane Database Syst Rev. 2014;(4):Cd001920. Published 2014 Apr 22. 10.1002/14651858.CD001920.pub3. 10.1002/14651858.CD001920.pub3 PMC6465059 24756870

[14] Unrath M, Kalic M, Berger K (2013). Who receives rehabilitation after stroke?: data from the quality assurance project "stroke register Northwest Germany". Deutsches Arzteblatt Int.

[15] Schupp W (1995). Konzept einer zustands- und behinderungsangepassten Behandlungs- und Rehabilitationskette in der neurologischen und neurochirurgischen Versorgung in Deutschland (“Phasenmodell”). Nervenarzt.

[16] Heck-Darabi. Hochschul-Befragung 2017 des Deutschen Verbandes für Physiotherapie 2017. Available from: https://www.physio-deutschland.de/fileadmin/data/bund/Dateien_oeffentlich/Beruf_und_Bildung/Studium/PHYSIO-DEUTSCHLAND_Hochschulbefragung_2017_Endfassung.pdf.

[17] Deutscher Verband für Physiotherapie (ZVK) e.V. Zahlen, Daten, Fakten zur Physiotherapie Köln: Physio Deutschland; 2021. https://www.physio-deutschland.de/fileadmin/data/bund/Dateien_oeffentlich/Beruf_und_Bildung/Zahlen__Daten__Fakten/Zahlen-Daten-Fakten-Juli21.pdf.

[18] Deutscher Verband für Physiotherapie (ZVK) e.V. Training and Examination Order for Physiotherapists (Physth-APrV) Köln: Physio Deutschland; 2012 [Available from: https://www.physiodeutschland.de/fileadmin/data/bund/Dateien_oeffentlich/Beruf_und_Bildung/Ausbildung/Training_and_examination_order.pdf.

[19] Bundesministerium für Justiz und Verbraucherschutz. Ausbildungs- und Prüfungsverordnung für Physiotherapeuten. Berlin: Bundesrepublik Deutschland; 2013. https://www.gesetze-im-internet.de/physth-aprv/BJNR378600994.html.

[20] Probst A. Versorgung von Menschen mit chronischen Schmerzen profitiert maßgeblich von der Akademisierung der Physiotherapie – die Fakten sprechen für sich. Schmerz. 2022. 10.1007/s00482-021-00615-9. 10.1007/s00482-021-00615-9 34981206

[21] Jolliffe L, Lannin NA, Cadilhac DA, Hoffmann T (2018). Systematic review of clinical practice guidelines to identify recommendations for rehabilitation after stroke and other acquired brain injuries. BMJ Open.

[22] Platz T (2019). Evidence-based guidelines and clinical pathways in stroke rehabilitation-an international perspective. Front Neurol.

[23] Kaendler S, Ritter M, Sander D, et al. Positionspapier Schlaganfallnachsorge der Deutschen Schlaganfall-Gesellschaft – Teil 1: Nachsorge nach einem Schlaganfall: Status quo der Versorgungsrealität und Versorgungsdefizite in Deutschland. Nervenarzt. 2022;93:368–76. 10.1007/s00115-021-01231-9. 10.1007/s00115-021-01231-9 PMC9010384 34978578

[24] Graham R, Mancher M, Wolman DM, Greenfield S, Steinberg E, Institute of Medicine (2011). Clinical practice guidelines we can trust.

[25] Duncan PW, Horner RD, Reker DM, Samsa GP, Hoenig H, Hamilton B (2002). Adherence to postacute rehabilitation guidelines is associated with functional recovery in stroke. Stroke..

[26] Pogrebnoy D, Dennett A (2020). Exercise programs delivered according to guidelines improve mobility in people with stroke: a systematic review and Meta-analysis. Arch Phys Med Rehabil.

[27] Ajimsha MS, Kooven S, Al-Mudahka N (2019). Adherence of physical therapy with clinical practice guidelines for the rehabilitation of stroke in an active inpatient setting. Disabil Rehabil.

[28] Johnston J, Mudge S, Kersten P, Jones A (2013). Physiotherapy alignment with guidelines for the management of stroke in the inpatient setting. N Z J Physiother.

[29] Hubbard IJ, Harris D, Kilkenny MF, Faux SG, Pollack MR, Cadilhac DA (2012). Adherence to clinical guidelines improves patient outcomes in Australian audit of stroke rehabilitation practice. Arch Phys Med Rehabil.

[30] Dohle, Quintern, Saal, Stephan, Tholen, Wittenberg. S2e-Leitlinie »Rehabilitation der Mobilität nach Schlaganfall (ReMoS)« Kurzfassung der Konsensusversion. Neurol Rehabil 2015;21(4):179–184.

[31] Pott C, Tiebel J (2019). Damit Leitlinien keine Leidlinien werden – Leitlinie Rehabilitation der Mobilität nach Schlaganfall (ReMoS). Physiopraxis..

[32] Dohle C, Tholen R, Wittenberg H, Quintern J, Saal S, Stephan KM (2016). Evidenzbasierte Rehabilitation der Mobilität nach Schlaganfall (Evidence-based rehabilitation of mobility after stroke) [German; with consumer summary]. Nervenarzt.

[33] DGNR, ZVK. ReMoS - Empfehlungen für die Therapie - Rehabilitation der Mobilität nach Schlaganfall 2020.

[34] Nelles G. et al. Rehabilitation von sensomotorischen Störungen, S2k-Leitlinie, 2018. Available from: https://www.awmf.org/uploads/tx_szleitlinien/030-123l_S2k_Rehabilitation_sensomotorische_St%C3%B6rungen_2018-04-verlaengert.pdf.

[35] Eysenbach G (2004). Improving the quality of web surveys: the checklist for reporting results of internet E-surveys (CHERRIES). J Med Internet Res.

[36] von Elm E, Altman DG, Egger M, Pocock SJ, Gotzsche PC, Vandenbroucke JP (2014). The strengthening the reporting of observational studies in epidemiology (STROBE) statement: guidelines for reporting observational studies. Int J Surg.

[37] Handley MA, Gorukanti A, Cattamanchi A (2016). Strategies for implementing implementation science: a methodological overview. Emerg Med J.

[38] Baker R, Camosso-Stefinovic J, Gillies C, Shaw EJ, Cheater F, Flottorp S, et al. Tailored interventions to address determinants of practice. Cochrane Database Syst Rev. 2015;(4):Cd005470. 10.1002/14651858.CD005470.pub3 PMC7271646 25923419

[39] Baker R, Camosso-Stefinovic J, Gillies C, Shaw EJ, Cheater F, Flottorp S, et al. Tailored interventions to overcome identified barriers to change: effects on professional practice and health care outcomes. Cochrane Database Syst Rev. 2010;(3):Cd005470. 10.1002/14651858.CD005470.pub2 PMC4164371 20238340

[40] Stander J, Grimmer K, Brink Y (2018). Training programmes to improve evidence uptake and utilisation by physiotherapists: a systematic scoping review. BMC Med Educ.

[41] Peters MAJ, Harmsen M, Laurant MGH, Wensing M (2002). Ruimte voor verandering? Knelpunten en mogelijkheden voor verandering in de patiëntenzorg [Room for improvement? Barriers to and facilitators for improvement of patient care].

[42] Dillman, Smyth, Christian. Internet, Phone, Mail, And mixed-mode surveys: the tailored design method: John Wiley & Sons; 2014.

[43] Willis GB, Artino AR (2013). What do our respondents think We're asking? Using cognitive interviewing to improve medical education surveys. J Graduate Med Educ.

[44] Bahns C, Happe L, Thiel C, Kopkow C (2021). Physical therapy for patients with low back pain in Germany: a survey of current practice. BMC Musculoskelet Disord.

[45] Spitaels D, Hermens R, Van Assche D, Verschueren S, Luyten F, Vankrunkelsven P (2017). Are physiotherapists adhering to quality indicators for the management of knee osteoarthritis? An observational study. Musculoskelet Sci Pract.

[46] Hasenbein U, Wallesch CW. What is "adherence to guidelines"? Theoretical and methodological considerations on a new concept for health system research and quality management. Gesundheitswesen (Bundesverband der Arzte des Offentlichen Gesundheitsdienstes (Germany). 2007;69(8–9):427–437. 10.1055/s-2007-985388 17926259

[47] Donohue A, McLaughlin C, Crowe M, Horgan F (2014). Clinical guideline adherence by physiotherapists working in acute stroke care. Ir Med J.

[48] PhysioDeutschland. Hochschulumfrage 2020 PHYSIO-DEUTSCHLAND ermittelt Absolventenzahlen von “Physiotherapeuten mit akademischem Abschluss” 2020. Available from: https://www.physiodeutschland.de/fileadmin/data/bund/Dateien_oeffentlich/Beruf_und_Bildung/Studium/Hochschulumfrage-2020.pdf.

[49] Grimshaw JM, Thomas RE, MacLennan G, Fraser C, Ramsay CR, Vale L, et al. Effectiveness and efficiency of guideline dissemination and implementation strategies. Health Technol Assess. 2004;8(6):iii-iv, 1–72. 10.3310/hta8060 14960256

[50] Donnellan C, Sweetman S, Shelley E (2013). Health professionals' adherence to stroke clinical guidelines: a review of the literature. Health Policy.

[51] Otterman NM, van der Wees PJ, Bernhardt J, Kwakkel G (2012). Physical therapists' guideline adherence on early mobilization and intensity of practice at dutch acute stroke units: a country-wide survey. Stroke..

[52] Flader CM, Rosendahl C, Gunther T (2017). Guideline conform diagnostics for dysphagia : a representative survey of speech therapists at certified stroke units in Germany. Nervenarzt..

[53] Tulek Z, Poulsen I, Gillis K, Jonsson AC (2018). Nursing care for stroke patients: a survey of current practice in 11 European countries. J Clin Nurs.

[54] Hammond R, Lennon S, Walker MF, Hoffman A, Irwin P, Lowe D (2005). Changing occupational therapy and physiotherapy practice through guidelines and audit in the United Kingdom. Clin Rehabil.

[55] Heinemann AW, Roth EJ, Rychlik K, Pe K, King C, Clumpner J (2003). The impact of stroke practice guidelines on knowledge and practice patterns of acute care health professionals. J Eval Clin Pract.

[56] Kolman AGJ. Guideline adherence in physical therapy: a systematic review: Utrecht University; 2012.

[57] Zadro J, O'Keeffe M, Maher C (2019). Do physical therapists follow evidence-based guidelines when managing musculoskeletal conditions? Systematic review. BMJ Open.

[58] McCluskey A, Ada L, Kelly PJ, Middleton S, Goodall S, Grimshaw JM (2015). Compliance with Australian stroke guideline recommendations for outdoor mobility and transport training by post-inpatient rehabilitation services: an observational cohort study. BMC Health Serv Res.

[59] Lynch EA, Connell LA, Carvalho LB, Bird ML. Do clinical guidelines guide clinical practice in stroke rehabilitation? An international survey of health professionals. Disabil Rehabil. 2021;1-8. 10.1080/09638288.2021.1891304 33651965

[60] Mirkowski M, Janzen S, Iruthayarajah J, Teasell R (2019). Factors influencing clinical guideline adherence in stroke rehabilitation care practices: a scoping review. Arch Phys Med Rehabil.

[61] Juckett LA, Wengerd LR, Faieta J, Griffin CE (2020). Evidence-based practice implementation in stroke rehabilitation: a scoping review of barriers and facilitators. Am J Occup Ther.

[62] Van Peppen RP, Maissan FJ, Van Genderen FR, Van Dolder R, Van Meeteren NL (2008). Outcome measures in physiotherapy management of patients with stroke: a survey into self-reported use, and barriers to and facilitators for use. Physiother Res Int.

[63] Gaskins NJ, Bray E, Hill JE, Doherty PJ, Harrison A, Connell LA. Factors influencing implementation of aerobic exercise after stroke: a systematic review. Disabil Rehabil. 2019:1–15. 10.1080/09638288.2019.1704075 31875459

[64] Moncion K, Biasin L, Jagroop D, Bayley M, Danells C, Mansfield A (2020). Barriers and facilitators to aerobic exercise implementation in stroke rehabilitation: a scoping review. J Neurol Phys Ther.

[65] Louie DR, Mortenson WB, Lui M, Durocher M, Teasell R, Yao J, et al. Patients' and therapists' experience and perception of exoskeleton-based physiotherapy during subacute stroke rehabilitation: a qualitative analysis. Disabil Rehabil. 2021;1-9. 10.1080/09638288.2021.1989503 34694189

[66] Bayley MT, Hurdowar A, Richards CL, Korner-Bitensky N, Wood-Dauphinee S, Eng JJ (2012). Barriers to implementation of stroke rehabilitation evidence: findings from a multi-site pilot project. Disabil Rehabil.

[67] McCluskey A, Vratsistas-Curto A, Schurr K (2013). Barriers and enablers to implementing multiple stroke guideline recommendations: a qualitative study. BMC Health Serv Res.

[68] Donnellan C, Sweetman S, Shelley E (2013). Implementing clinical guidelines in stroke: a qualitative study of perceived facilitators and barriers. Health Policy.

[69] Mudge S, Hart A, Murugan S, Kersten P (2017). What influences the implementation of the New Zealand stroke guidelines for physiotherapists and occupational therapists?. Disabil Rehabil.

[70] Munce SEP, Graham ID, Salbach NM, Jaglal SB, Richards CL, Eng JJ (2017). Perspectives of health care professionals on the facilitators and barriers to the implementation of a stroke rehabilitation guidelines cluster randomized controlled trial. BMC Health Serv Res.

[71] Nelson ML, Grudniewicz A, Albadry S (2016). Applying clinical practice guidelines to the complex patient: insights for practice and policy from stroke rehabilitation. Healthc Q.

[72] Jolliffe L, Hoffmann T, Lannin NA. Increasing the uptake of stroke upper limb guideline recommendations with occupational therapists and physiotherapists. A qualitative study using the Theoretical Domains Framework. Aust Occup Ther J. 2019. 10.1111/1440-1630.12599 31338859

[73] Alatawi SF (2019). From theory to practice: a conceptual framework to facilitate implementation of evidence in stroke rehabilitation for local context in Saudi Arabia. J Multidiscip Healthc.

[74] Physiotherapy. WPGAf. 2021 Available from: world Physiotherapy. German Association for Physiotherapy.

[75] Braun T, Rieckmann A, Weber F, Gruneberg C (2018). Current use of measurement instruments by physiotherapists working in Germany: a cross-sectional online survey. BMC Health Serv Res.

[76] Kristensen HK, Ytterberg C, Jones DL, Lund H (2016). Research-based evidence in stroke rehabilitation: an investigation of its implementation by physiotherapists and occupational therapists. Disabil Rehabil.

[77] Cahill LS, Carey LM, Lannin NA, Turville M, Neilson CL, Lynch EA, et al. Implementation interventions to promote the uptake of evidence-based practices in stroke rehabilitation. Cochrane Database Syst Rev. 2020;10. 10.1002/14651858.CD012575.pub2 PMC8095062 33058172

[78] Connell L, Chesworth B, Lynch E (2018). 62 implementation: the missing link in the stroke rehabilitation research pipeline. BMJ Evid Based Med.

[79] Gagliardi AR, Marshall C, Huckson S, James R, Moore V (2015). Developing a checklist for guideline implementation planning: review and synthesis of guideline development and implementation advice. Implement Sci.

