# Supplementary figures and images for: Current physical therapy practice and implementation factors regarding the evidence-based ‘Rehabilitation of Mobility after Stroke (ReMoS)’ guideline in Germany: a cross-sectional online survey
Source: BMC Neurol. 2022 Jul 30;22:284. doi: 10.1186/s12883-022-02780-5 (PMC9338587; doi:10.1186/s12883-022-02780-5)

Additional file 1.

Process of guideline development according to Dohle et al., 2016 (32).

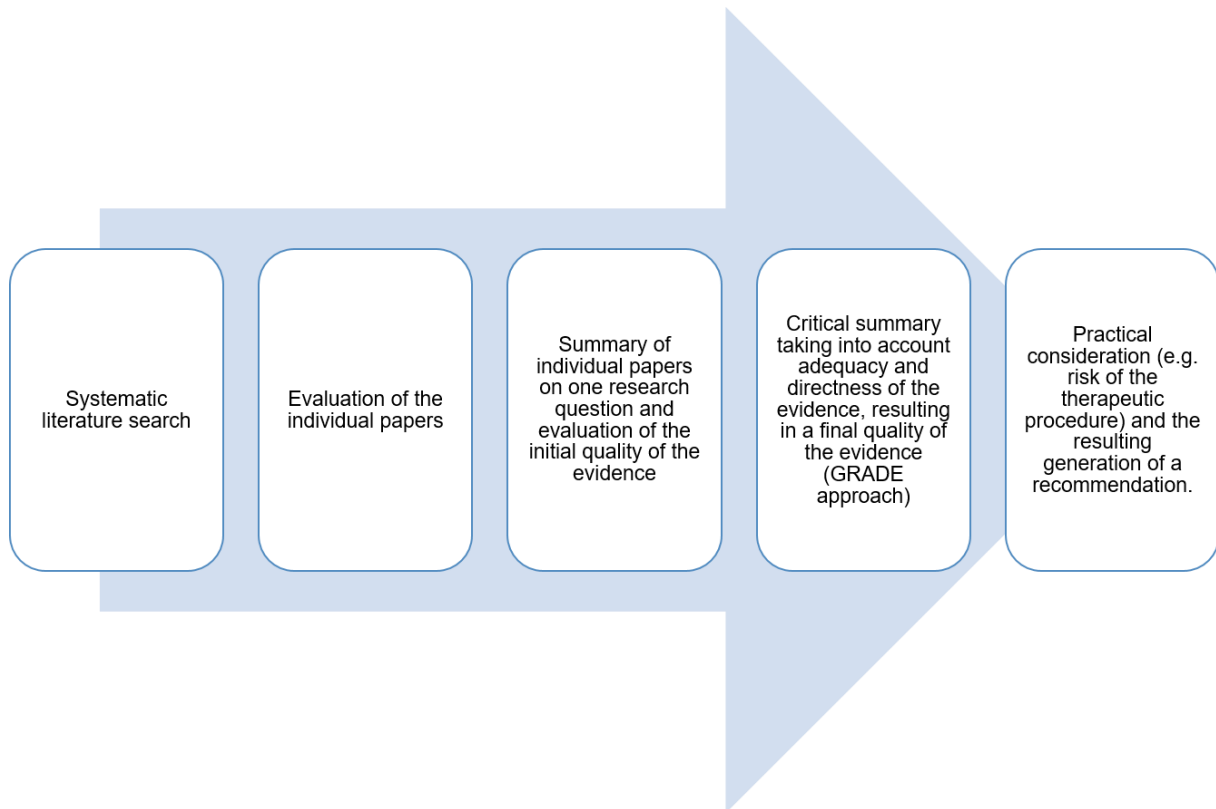

Supplement: Supplementary file 1 — Additional file 1. Process of guideline development according to Dohle et al., 2016 [32]. [file 12883_2022_2780_MOESM1_ESM.pdf]
