# Supplementary material for: Current physical therapy practice and implementation factors regarding the evidence-based ‘Rehabilitation of Mobility after Stroke (ReMoS)’ guideline in Germany: a cross-sectional online survey
Source: BMC Neurol. 2022 Jul 30;22:284. doi: 10.1186/s12883-022-02780-5 (PMC9338587; doi:10.1186/s12883-022-02780-5)
Supplement: Supplementary file 2 — Additional file 2. Original German version of the questionnaire. [file 12883_2022_2780_MOESM2_ESM.pdf]

Sehr geehrte Physiotherapeut*Innen,

Ihre Arbeit fördert nachweislich die Mobilität von Menschen nach einem Schlaganfall.

In dieser deutschlandweiten Umfrage wird erfasst, welche Maßnahmen Physiotherapeut*Innen auswählen, um die Gehfähigkeit, Gehgeschwindigkeit, Gehstrecke und Balance in der subakuten Phase nach einem Schlaganfall zu verbessern.

Wir möchten überprüfen, inwieweit die Maßnahmen den Empfehlungen der S2e-Leitlinie "Rehabilitation der Moblität nach Schlaganfall (ReMoS)" entsprechen und erfahren, welche Faktoren die Umsetzung der Leitlinienempfehlungen beeinflussen.

Die Ergebnisse können dazu beitragen zu verstehen, ob Menschen nach einem Schlaganfall leitliniengerechte Therapie erfahren und auf welcher Grundlage Physiotherapeut*Innen ihre Entscheidungen zum Therapieprozess treffen.

Hinweise zur Befragung

Wir bitten Sie die Fragen zur aktuellen Ausgestaltung Ihrer Therapieeinheiten von Menschen in der subakuten Phase nach einem Schlaganfall sowie Fragen zu Ihren demografischen Angaben zu beantworten. Der Fragebogen umfasst maximal 89 Fragen und wird etwa 15 - 20 Minuten Ihrer Zeit in Anspruch nehmen. Bitte antworten Sie gemäß Ihrer eigenen, aktuellen beruflichen Erfahrungen aus der Rehabilitation von Menschen nach einem Schlaganfall.

Bitte lesen die Begriffserläuterungen, die den Fragen als Hilfstexte beigefügt sind. Sie sollen dem einheitlichen Verständnis und der konkreten Beantwortung der Fragen dienen. Nutzen Sie die Möglichkeit Ihre Antworten durch einen Freitext zu ergänzen.

Zusätzlich würden wir uns freuen, wenn Sie Ihre Bereitschaft zur Teilnahme an weiteren Erhebungen zu diesem Forschungsvorhaben erklären und dafür Ihre E-Mail-Adresse hinterlassen.

Danke, dass Sie uns bei diesem Forschungsvorhaben unterstützen!

Gern können Sie Ihre Kolleg*Innen zur Teilnahme einladen und den Link zur Befragung an sie weiterleiten.

Teil A: Bestätigung der Zugangsvoraussetzungen

Bitte bestätigen Sie, sofern zutreffend, die Zugangsvoraussetzungen bevor Sie mit der freiwilligen Befragung beginnen.

A1. Sie sind ein*e staatlich anerkannte*r Physiotherapeut*In und behandeln derzeit Menschen in der subakuten Erkrankungsphase nach einem Schlaganfall in Deutschland?

Die subakute Phase nach einem Schlaganfall bezeichnet die Zeit ab der 4. Woche bis zum 6. Monat nach dem Ereignis.

Ja ☐

Nein ☐

Teil B: Maßnahmen zur Verbesserung der Gehfähigkeit von initial nicht-gehfähigen Patient*Innen

Wie häufig nutzen Sie folgende Interventionen um die Gehfähigkeit von initial nichtgehfähigen Menschen nach einem Schlaganfall zu erreichen?

B1. Intensives Gehtraining

Intensives Gehtraining meint das Üben einzelner Gangphasen oder des gesamten Gangzyklus auf dem Boden bei einer Intensität von mindestens 30 Minuten am Tag.

Immer ☐

Oft ☐

Manchmal ☐

Nie ☐

B2. Intensives Gehtraining unter Einschluss Endeffektor-basierter Geräte (z. B. Gangtrainer)

Intensives Gehtraining meint das Üben einzelner Gangphasen oder des gesamten Gangzyklus auf dem Boden bei einer Intensität von mindestens 30 Minuten am Tag.

*Gangtrainer sind elektromechanische endeffektor-basierte Trainingsgeräte, die über bewegliche Fußplatten den Gangzyklus trainieren. Die Patient*Innen werden durch Gurtsysteme unterstützt und gesichert.*

Immer ☐

Oft ☐

Manchmal ☐

Nie ☐

B3. Intensives Gehtraining unter Einschluss des Laufbands oder eines Exoskelettgestützten Gerätes (z.B. Lokomat)

Intensives Gehtraining meint das Üben einzelner Gangphasen oder des gesamten Gangzyklus bei einer Intensität von mindestens 30 Minuten am Tag.

Lokomaten sind Exoskelett-gestützte Trainingsgeräte zum Training des Gangzyklus mit Führung der Hüften und Beine.

Immer ☐

Oft ☐

Manchmal ☐

Nie ☐

B4. Zyklische Mehrkanalstimulation zur Erzeugung gehähnlicher Beinbewegungendes paretischen Beines im Liegen

Zyklische Mehrkanalstimulation meint eine Form der elektrischen Stimulation zur Erzeugung gangähnlicher Bewegungen des in Seitlage hängenden paretischen Beines.

- Immer ☐
- Oft ☐
- Manchmal ☐
- Nie ☐

B5. Um die Gehfähigkeit von Menschen nach einem Schlaganfall zur erreichen, die initial nicht gehfähig sind nutze ich andere Interventionen. Diese sind:

Teil C: Maßnahmen zur Verbesserung der Gehfähigkeit von (eingeschränkt) gehfähigen Patient*Innen

Wie häufig nutzen Sie folgende Interventionen um die Gehfähigkeit von (eingeschränkt) gehfähigen Menschen nach einem Schlaganfall zu verbessern?

C1. Intensives konventionelles Gehtraining

Intensives konventionelles Gehtraining meint das Üben einzelner Gangphasen oder des gesamten Gangzyklus auf dem Boden bei einer Intensität von mindestens 30 Minuten am Tag.

- Immer ☐
- Oft ☐
- Manchmal ☐
- Nie ☐

C2. Intensives und möglichst progressives Gehtraining unter Einschluss des Laufbandes

Intensives Gehtraining meint das Üben einzelner Gangphasen oder des gesamten Gangzyklus bei einer Intensität von mindestens 30 Minuten am Tag.

Progressiv meint die kontinuierliche Steigerung der Trainingsanforderung durch Erhöhung der Wiederholungsanzahl oder Erhöhung des Schwierigkeitsgrades einzelner Bewegungsaufgaben.

- Immer ☐
- Oft ☐
- Manchmal ☐
- Nie ☐

LimeSurvey

C3. Aufgabenbezogenes Training mit Bewegungsvorstellung

Aufgabenbezogenes Training meint die Verbesserung der Bewegungsausführung mittels funktioneller Bewegungsübungen.

Bewegungsvorstellung (mentales Training) ist die gedankliche Vorstellung von Bewegungen des eigenen Körpers oder eigener Körperteile, ohne diese aktiv auszuführen.

- Immer ☐
- Oft ☐
- Manchmal ☐
- Nie ☐

C4. Nutzung von Gehhilfen

Gehhilfen sind Hilfsmittel zur direkten oder indirekten Unterstützung des Gehtrainings oder der Balance, z.B. Gehstock, Sprunggelenksorthese, Gewichtsmanschetten, Armschlingen, Taping.

- Immer ☐
- Oft ☐
- Manchmal ☐
- Nie ☐

C5. Kombinationstherapie aus Endeffektor-basiertem Gerät (z.B. Gangtrainer) mit funktioneller Elektrostimulation

Kombinationstherapie meint das Gangtraining unter Anwendung eines Gangtrainers bei zeitgleicher Elektrostimulation des Musculus quadriceps femoris (zur Kniestabilisation während der Standbeinphase) sowie des Nervus peronaeus (Aktivierung der Dorsalextension während der Schwungbeinphase).

- Immer ☐
- Oft ☐
- Manchmal ☐
- Nie ☐

C6. Um die Gehfähigkeit von (eingeschränkt) gehfähigen Menschen nach einem Schlaganfall zur verbessern nutze ich andere Interventionen. Diese sind:

☐

Teil D: Maßnahmen zur Verbesserung der Gehgeschwindigkeit von (eingeschränkt) gehfähigen Patient*Innen

Wie häufig nutzen Sie folgende Interventionen um die Gehgeschwindigkeit von (eingeschränkt) gehfähigen Menschen nach einem Schlaganfall zu verbessern?

D1. Aufgabenbezogenes Ausdauertraining mittels progressivem Laufbandtraining

Aufgabenbezogenes Training meint die Verbesserung der Bewegungsausführung mittels funktioneller Bewegungsübungen.

Progressiv meint die kontinuierliche Steigerung der Trainingsanforderung durch Erhöhung der Wiederholungsanzahl oder Erhöhung des Schwierigkeitsgrades einzelner Bewegungsaufgaben.

- Immer ☐
- Oft ☐
- Manchmal ☐
- Nie ☐

D2. Aufgabenbezogenes Ausdauertraining mittels progressivem Zirkeltraining

Aufgabenbezogenes Training meint die Verbesserung der Bewegungsausführung mittels funktioneller Bewegungsübungen.

Progressiv meint die kontinuierliche Steigerung der Trainingsanforderung durch Erhöhung der Wiederholungsanzahl oder Erhöhung des Schwierigkeitsgrades einzelner Bewegungsaufgaben.

- Immer ☐
- Oft ☐
- Manchmal ☐
- Nie ☐

D3. Intensives Gehtraining ohne Laufband

Intensives Gehtraining meint das Üben einzelner Gangphasen oder des gesamten Gangzyklus auf dem Boden bei einer Intensität von mindestens 30 Minuten am Tag.

- Immer ☐
- Oft ☐
- Manchmal ☐
- Nie ☐

D4. Intensives Gehtraining unter Einschluss des Laufbands

Intensives Gehtraining meint das Üben einzelner Gangphasen oder des gesamten Gangzyklus auf dem Laufband bei einer Intensität von mindestens 30 Minuten am Tag.

- Immer ☐
- Oft ☐
- Manchmal ☐
- Nie ☐

D5. Intensives, supervidiertes, progressives Heimübungsprogramm (Kräftigung, Ausdauer, Balance)

Intensiv meint das Training über mehrere Wochen.

Supervidiertes Training meint die Beratung und Beaufsichtigung der Übungseinheiten durch einen Physiotherapeuten in regelmäßigen Abständen.

Progressiv meint die kontinuierliche Steigerung der Trainingsanforderung durch Erhöhung der Wiederholungsanzahl oder Erhöhung des Schwierigkeitsgrades einzelner Bewegungsaufgaben.

Immer ☐
Oft ☐
Manchmal ☐
Nie ☐

D6. Gangtraining mit Stimulation von Flexorreflex-Afferenzen

Die Stimulation der Flexorreflex-Afferenzen meint die sensorische Elektrostimulation des paretischen Beines mit Oberflächenelektroden während des Gehens. Schrittsynchron werden so Beugesynergien in Hüft- und Kniegelenk sowie die Dorsalextension im Sprunggelenk erzeugt. Dies unterstützt die Schwungbeinphase im Gangzyklus.

Immer ☐
Oft ☐
Manchmal ☐
Nie ☐

D7. Zusätzliches Beinfunktionstraining

Zusätzliches Zusätzlich meint die Durchführung des Trainings in Ergänzung zur Standardtherapie.

Beinfunktionstraining meint ein spezifisches Training für die untere Extremität.

Immer ☐
Oft ☐
Manchmal ☐
Nie ☐

D8. Intensives progressives aufgabenbezogenes Training

Progressiv meint die kontinuierliche Steigerung der Trainingsanforderung durch Erhöhung der Wiederholungsanzahl oder Erhöhung des Schwierigkeitsgrades einzelner Bewegungsaufgaben.

Aufgabenbezogenes Training ist die Auswahl der Bewegungsübungen aufgrund der tatsächlichen Aktivitäten des täglichen Lebens.

Immer ☐
Oft ☐
Manchmal ☐
Nie ☐

LimeSurvey

D9. Aufgabenbezogenes Training mit Bewegungsvorstellung

Aufgabenbezogenes Training meint die Verbesserung der Bewegungsausführung mittels funktioneller Bewegungsübungen.

Bewegungsvorstellung (mentales Training) meint die gedankliche Vorstellung von Bewegungen des eigenen Körpers oder eigener Körperteile, ohne diese aktiv auszuführen.

Immer ☐
Oft ☐
Manchmal ☐
Nie ☐

D10. Gehtraining mit einem Endeffektor-basiertem Gerät (z.B. Gangtrainer) oder Gehtraining mit einem Exoskelett-gestütztem Gerät (z.B. Lokomat)

*Gangtrainer sind elektromechanische endeffektor-basierte Trainingsgeräte, die über bewegliche Fußplatten den Gangzyklus trainieren. Die Patient*Innen werden durch Gurtsysteme unterstützt und gesichert.*

Lokomaten sind Exoskelet-gestützte Trainingsgeräte zum Training des Gangzyklus mit Führung der Hüften und Beine.

☐
Immer ☐
Oft ☐
Manchmal ☐
Nie ☐

D11. Kraft-Ausdauertraining

Immer ☐
Oft ☐
Manchmal ☐
Nie ☐

D12. Isokinetisches Krafttraining

Isokinetisches Krafttraining meint die Anwendung eines kontinuierlichen Wiederstandes während der gesamten Bewegungsamplitude.

Immer ☐
Oft ☐
Manchmal ☐
Nie ☐

D13. Gehtraining mit akustischer Stimulation

Gehtraining meint das Üben einzelner Gangphasen oder des gesamten Gangzyklus auf dem Boden.

Akustische Stimulation meint einen hörbaren externen Taktgeber (z.B. Metronom) zur Verbesserung der motorischen Kontrolle in den Bewegungsabläufen.

Immer ☐
Oft ☐
Manchmal ☐
Nie ☐

D14. Akustisches Feedback beim Gehen

Akustisches Feedback meint ein hörbares Signal als Rückmeldung über die Bewegungsqualität oder den Bewegungserfolg zur optimierung des Lernprozesses.

Immer ☐
Oft ☐
Manchmal ☐
Nie ☐

D15. Feedback / Reinforcement (tägliche Zeitmessung beim Gehen mit verstärkendem Feedback)

Feedback und Reinforcement meinen eine spezifische, verstärkende verbale Rückmeldung zur Gehgeschwindigkeit, um den Lernprozess zu optimieren.

Immer ☐
Oft ☐
Manchmal ☐
Nie ☐

D16. Kombinationstherapie aus Endeffektor-basiertem Gerät (z.B. Gangtrainer) mit funktioneller Elektrostimulation

Kombinationstherapie meint das Gangtraining unter Anwendung eines Gangtrainers bei zeitgleicher Elektrostimulation des Musculus quadriceps femoris (zur Kniestabilisation während der Standbeinphase) sowie des Nervus peronaeus (Aktivierung der Dorsalextension während der Schwungbeinphase).

Immer ☐
Oft ☐
Manchmal ☐
Nie ☐

D17. Nutzung von Sprunggelenksorthesen

Sprunggelenksorthesen dienen der Korrektor von Fehlhaltung des Fußes aufgrund einer Fußheberschwäche und ermöglichen damit einen festeren Halt beim Gehen.

Immer ☐
Oft ☐
Manchmal ☐
Nie ☐

LimeSurvey

D18. Frühzeitige Nutzung von orthopädischen Schuhen

Tämporärer Einsatz von orthopädischen Schuhen am Anfang der Rehabilitation.

- Immer ☐
- Oft ☐
- Manchmal ☐
- Nie ☐

D19. Nutzung von Armschlingen bei schweren Armparesen

- Immer ☐
- Oft ☐
- Manchmal ☐
- Nie ☐

D20. Um die die Gehgeschwindigkeit von (eingeschränkt) gehfähigen Menschen nach einem Schlaganfall zu verbessern nutze ich andere Interventionen. Diese sind:

Teil E: Maßnahmen zur Verbesserung der Gehstrecke von (eingeschränkt) gehfähigen Patient*Innen

Wie häufig nutzen Sie folgende Interventionen um die Gehstrecke von (eingeschränkt) gehfähigen Menschen nach einem Schlaganfall zu erweitern?

E1. Aufgabenbezogenes Ausdauertraining

Aufgabenbezogenes Training meint die Verbesserung der Bewegungsausführung mittels funktioneller Bewegungsübungen.

- Immer ☐
- Oft ☐
- Manchmal ☐
- Nie ☐

E2. Intensives, supervidiertes, progressives Heimübungsprogramm (Kräftigung, Audauer, Balance)

Supervidiertes Training meint die Beratung und Beaufsichtigung durch einen Physiotherapeuten während einer Übungseinheit.

Progressiv meint die kontinuierliche Steigerung der Trainingsanforderung durch Erhöhung der Wiederholungsanzahl oder Erhöhung des Schwierigkeitsgrades einzelner Bewegungsaufgaben.

- Immer ☐
- Oft ☐
- Manchmal ☐
- Nie ☐

LimeSurvey

E3. Intensives Gehtraining unter Einschluss des Laufbands; insbesondere progressives, aerobes Laufbandtraining

Intensives Gehtraining meint das Üben einzelner Gangphasen oder des gesamten Gangzyklus bei einer Intensität von mindestens 30 Minuten am Tag.

Progressiv meint die kontinuierliche Steigerung der Trainingsanforderung durch Erhöhung der Wiederholungsanzahl oder Erhöhung des Schwierigkeitsgrades einzelner Bewegungsaufgaben. Aerobes Training meint entsprechend der Kriterien der American College of Sports Medicine (ACSM) und der American Heart Association/American Stroke Association (AHA/ASA) ein 20 - 60 minütiges Training der Ausdauer an 3 bis 7 Tagen in der Woche. Die Belastungsintensität sollte dabei zwischen 50 - 80 % der maximalen Herzfrequenz liegen. Alternativ sollte die Belastung einem subjektiven Empfinden von 11 bis 14 Punkten auf einer Skala zum subjektiven Belastungsempfinden (6-20 Punkte) entsprechen.

Immer ☐
Oft ☐
Manchmal ☐
Nie ☐

E4. Aufgabenbezogenes Training mit Bewegungsvorstellung

Aufgabenbezogenes Training meint die Verbesserung der Bewegungsausführung mittels funktioneller Bewegungsübungen.

Bewegungsvorstellung (mentales Training) ist die gedankliche Vorstellung von Bewegungen des eigenen Körpers oder eigener Körperteile, ohne diese aktiv auszuführen.

Immer ☐
Oft ☐
Manchmal ☐
Nie ☐

E5. Gehtraining mit einem Endeffektor-basiertem Gerät (z. B. Gangtrainer) oder Gehtraining mit einem Exoskelett-gestütztem Gehtrainer (z.B. Lokomat)

Gangtrainer sind elektromechanische endeffektor-basierte Trainingsgeräte, die über bewegliche Fußplatten den Gangzyklus trainieren.

Lokomaten sind Exoskelet-gestützte Trainingsgeräte zum Training des Gangzyklus mit Führung der Hüften und Beine.

Immer ☐
Oft ☐
Manchmal ☐
Nie ☐

E6. Kraft-Ausdauertraining

Immer ☐
Oft ☐
Manchmal ☐
Nie ☐

E7. Funktionelle Elektrische Mehrkanalstimulation während des Gangtrainings

Funktionelle Elektrische Mehrkanalstimulation meint die selektive schrittsynchrone Stimulation mehrerer Muskeln/ Muskelgruppen des paretischen Beines während des Gehens.

- Immer ☐
- Oft ☐
- Manchmal ☐
- Nie ☐

E8. Hochfrequente Physiotherapie im häuslichen Umfeld

- Immer ☐
- Oft ☐
- Manchmal ☐
- Nie ☐

E9. Zusätzliches Kraft-Ausdauertraining

Zusätzlich meint die Durchführung des Trainings in Ergänzung zur regulären Therapie.

- Immer ☐
- Oft ☐
- Manchmal ☐
- Nie ☐

E10. Zusätzliches Beinfunktionstraining

Beinfunktionstraining meint ein spezifisches Training für die untere Extremität

Zusätzlich meint die Durchführung des Trainings in Ergänzung zur Standardtherapie.

- Immer ☐
- Oft ☐
- Manchmal ☐
- Nie ☐

E11. Um die Gehstrecke von (eingeschränkt) gehfähigen Menschen nach einem Schlaganfall zu erweitern nutze ich andere Interventionen. Diese sind:

Teil F: Maßnahmen zur Sturzvermeidung und zur Verbesserung der statischen und dynamischen Balance

Wie häufig nutzen Sie folgende Interventionen um die statische und dynamische Balance von Menschen nach einem Schlaganfall zu verbessern und Stürze zu verhindern?

F1. Intensives Gehtraining ohne Laufband

Intensives Gehtraining meint das Üben einzelner Gangphasen oder des gesamten Gangzyklus auf dem Boden bei einer Intensität von mindestens 30 Minuten am Tag.

- Immer ☐
- Oft ☐
- Manchmal ☐
- Nie ☐

F2. Intensives Gehtraining unter Einschluss des Laufbands

Intensives Gehtraining meint das Üben einzelner Gangphasen oder des gesamten Gangzyklus auf dem Laufband bei einer Intensität von mindestens 30 Minuten am Tag.

- Immer ☐
- Oft ☐
- Manchmal ☐
- Nie ☐

F3. Intensives, supervidiertes, progressives Heimübungsprogramm (Kräftigung, Ausdauer, Balance)

Supervidiertes Training meint die Beratung und Beaufsichtigung durch einen Physiotherapeuten während einer Übungseinheit.

Progressiv meint die kontinuierliche Steigerung der Trainingsanforderung durch Erhöhung der Wiederholungsanzahl oder Erhöhung des Schwierigkeitsgrades einzelner Bewegungsaufgaben.

- Immer ☐
- Oft ☐
- Manchmal ☐
- Nie ☐

F4. Motor Relearning Programm

Das Motor Relearning Programm meint das aufgabenorientierte Erlernen von Aktivitäten des täglichen Lebens zur Verbesserung der motorischen Funktionen.

- Immer ☐
- Oft ☐
- Manchmal ☐
- Nie ☐

F5. Gangtraining mit einem Laufband, einem Endeffektor-basiertem Gerät (z.B. Gangtrainer) oder einem Exoskelett-gestütztem Gerät (z.B. Lokomat)

*Gangtrainer sind elektromechanische endeffektor-basierte Trainingsgeräte, die über bewegliche Fußplatten den Gangzyklus trainieren. Die Patient*Innen werden durch Gurtsysteme unterstützt und gesichert.*

Lokomaten sind Exoskelet-gestützte Trainingsgeräte zum Training des Gangzyklus mit Führung der Hüften und Beine.

- Immer ☐
- Oft ☐
- Manchmal ☐
- Nie ☐

F6. Kraft- Ausdauertraining

- Immer ☐
- Oft ☐
- Manchmal ☐
- Nie ☐

F7. Rumpfaktivitäten auf instabiler Unterstützungsfläche

- Immer ☐
- Oft ☐
- Manchmal ☐
- Nie ☐

F8. Akustisches Feedback beim Gehen

Akustisches Feedback meint ein hörbares externes Signal als Rückmeldung über die Bewegungsqualität oder den Bewegungserfolg.

- Immer ☐
- Oft ☐
- Manchmal ☐
- Nie ☐

F9. Frühzeitige Nutzung von orthopädischen Schuhen

Temporärer Einsatz von orthopädischen Schuhen am Anfang der Rehabilitation.

- Immer ☐
- Oft ☐
- Manchmal ☐
- Nie ☐

LimeSurvey

F10. Zusätzliches Ergometertraining

Zusätzlich meint die Durchführung des Trainings in Ergänzung zum regulären Rehabilitationsprogramm.

Immer ☐
Oft ☐
Manchmal ☐
Nie ☐

F11. Zusätzliches Training auf einer Bio-Feedbackplattform mit erweiterten Aufgabenstellungen

Zusätzlich meint die Durchführung des Trainings in Ergänzung zur konventionellen Therapie.

Bio-Feedback-Plattformen meinen Kraftmessplatten, die über einen Monitor Rückmeldungen zur Position des Körperschwerpunktes geben, um den Lernprozess zu unterstützen.

Immer ☐
Oft ☐
Manchmal ☐
Nie ☐

F12. Um die statische und dynamische Balance von Menschen nach einem Schlaganfall zu verbessern und Stürze zu verhindern nutze ich andere Interventionen. Diese sind:

Teil G: S2-e Leitlinie "Rehabilitation der Mobilität nach Schlaganfall"

Ich danke Ihnen für die Beantwortung der Fragen bis hierher! Bitte beantworten Sie nun noch Fragen zu Ihren persönlichen Erfahrungen mit Leitlinien.

G1. Ich kenne die S2e-Leitlinie „Rehabilitation der Mobilität nach Schlaganfall (ReMoS)“.

Ja ☐
Nein ☐

G2. Ich habe über folgenden Weg von der ReMoS-Leitlinie erfahren:

Teil H: Barrieren und Förderfaktoren zur Umsetzung von Leitlinienempfehlungen

H1. **Die ReMoS-Leitlinie ermöglicht es mir meine eigenen Entscheidungen zu treffen.**

- Trifft voll und ganz zu ☐
- Trifft ziemlich zu ☐
- Trifft teilweise zu ☐
- Trifft wenig zu ☐
- Trifft überhaupt nicht zu ☐

H2. **Die ReMoS-Leitlinie ermöglicht es mir die Präferenzen der Patient*Innen zu berücksichtigen.**

- Trifft voll und ganz zu ☐
- Trifft ziemlich zu ☐
- Trifft teilweise zu ☐
- Trifft wenig zu ☐
- Trifft überhaupt nicht zu ☐

H3. **Die ReMoS-Leitlinie stellt eine gute Grundlage für meine eigene Bildung dar.**

- Trifft voll und ganz zu ☐
- Trifft ziemlich zu ☐
- Trifft teilweise zu ☐
- Trifft wenig zu ☐
- Trifft überhaupt nicht zu ☐

H4. **Ich habe die Empfehlungen der ReMoS-Leitlinie nicht detailliert durchgearbeitet.**

- Trifft voll und ganz zu ☐
- Trifft ziemlich zu ☐
- Trifft teilweise zu ☐
- Trifft wenig zu ☐
- Trifft überhaupt nicht zu ☐

H5. **Ich möchte gern mehr über die ReMoS-Leitlinie wissen, bevor ich mich dafür entscheide die Empfehlungen anzunehmen.**

- Trifft voll und ganz zu ☐
- Trifft ziemlich zu ☐
- Trifft teilweise zu ☐
- Trifft wenig zu ☐
- Trifft überhaupt nicht zu ☐

H6. **Ich habe Schwierigkeiten damit meine alten Gewohnheiten abzulegen.**

- Trifft voll und ganz zu ☐
- Trifft ziemlich zu ☐
- Trifft teilweise zu ☐
- Trifft wenig zu ☐
- Trifft überhaupt nicht zu ☐

H7. **Ich denke, dass Aspekte der ReMoS-Leitlinie nicht korrekt sind.**

- Trifft voll und ganz zu ☐
- Trifft ziemlich zu ☐
- Trifft teilweise zu ☐
- Trifft wenig zu ☐
- Trifft überhaupt nicht zu ☐

H8. **Ich mag es generell nicht nach Vorschriften zu arbeiten.**

- Trifft voll und ganz zu ☐
- Trifft ziemlich zu ☐
- Trifft teilweise zu ☐
- Trifft wenig zu ☐
- Trifft überhaupt nicht zu ☐

H9. **Meine Kolleg*Innen aus dem Bereich Pflege und Physiotherapie wirken an der Umsetzung der ReMoS-Leitlinie nicht mit.**

- Trifft voll und ganz zu ☐
- Trifft ziemlich zu ☐
- Trifft teilweise zu ☐
- Trifft wenig zu ☐
- Trifft überhaupt nicht zu ☐

LimeSurvey

H10. Neurolog*Innen und/ oder andere Ärzt*Innen wirken an der Umsetzung der ReMoS-Leitlinie nicht mit.

- Trifft voll und ganz zu ☐
- Trifft ziemlich zu ☐
- Trifft teilweise zu ☐
- Trifft wenig zu ☐
- Trifft überhaupt nicht zu ☐

H11. Meine Vorgesetzten wirken an der Umsetzung der ReMoS-Leitlinie nicht mit.

- Trifft voll und ganz zu ☐
- Trifft ziemlich zu ☐
- Trifft teilweise zu ☐
- Trifft wenig zu ☐
- Trifft überhaupt nicht zu ☐

H12. Die Patient*Innen wirken an der Umsetzung der ReMoS-Leitlinie nicht mit.

- Trifft voll und ganz zu ☐
- Trifft ziemlich zu ☐
- Trifft teilweise zu ☐
- Trifft wenig zu ☐
- Trifft überhaupt nicht zu ☐

H13. Die Arbeit mit der ReMoS-Leitlinie beansprucht zu viel Zeit.

- Trifft voll und ganz zu ☐
- Trifft ziemlich zu ☐
- Trifft teilweise zu ☐
- Trifft wenig zu ☐
- Trifft überhaupt nicht zu ☐

H14. Die Arbeit mit der ReMoS-Leitlinie widerspricht meiner täglichen beruflichen Praxis.

- Trifft voll und ganz zu ☐
- Trifft ziemlich zu ☐
- Trifft teilweise zu ☐
- Trifft wenig zu ☐
- Trifft überhaupt nicht zu ☐

H15. Die Arbeit nach der ReMoS-Leitlinie erfordert einen finziellen Ausgleich.

- Trifft voll und ganz zu ☐
- Trifft ziemlich zu ☐
- Trifft teilweise zu ☐
- Trifft wenig zu ☐
- Trifft überhaupt nicht zu ☐

H16. Das Layout der ReMoS-Leitlinie ist in der täglichen Arbeit einfach zu benutzen.

- Trifft voll und ganz zu ☐
- Trifft ziemlich zu ☐
- Trifft teilweise zu ☐
- Trifft wenig zu ☐
- Trifft überhaupt nicht zu ☐

H17. Meiner Meinung nach bedingen noch andere Faktoren die Umsetzung der ReMoS-Leitlinie. Diese sind:

- Verfügbarkeit eines Endeffektor-basierten Gerätes (z.B. Gangtrainer) ☐
- Verfügbarkeit eins Exoskellett-basierten Gerätes (z.B. Lokomat) ☐
- Verfügbarkeit eines Laufbands ☐
- Kenntnis über empfohlene Interventionen ☐
- Beteiligung anderer Berufsgruppen (z.B. Sporttherapeut*Innen, Masseur*Innen und Medizinische Bademeister*Innen, ...) ☐
- Fehlender Bezug zum Phasenmodell der neurologischen Rehabilitation ☐
- Andere ☐

LimeSurvey

Teil I: Soziodemographische Angaben

Die Befragung ist fast beendet. Ich bitte Sie nun um die Angaben zu Ihrer Person und Ihrer Arbeit als Physiotherapeut*In.

I1. Wie alt sind Sie?

☐☐

I2. Welches Geschlecht haben Sie?

- weiblich ☐
- männlich ☐

I3. In welchem Bundesland arbeiten Sie?

- Baden-Württemberg ☐
- Bayern ☐
- Berlin ☐
- Brandenburg ☐
- Bremen ☐
- Hamburg ☐
- Hessen ☐
- Mecklenburg-Vorpommern ☐
- Niedersachsen ☐
- Nordrhein-Westfalen ☐
- Rheinland-Pfalz ☐
- Saarland ☐
- Sachsen ☐
- Sachsen-Anhalt ☐
- Schleswig-Holstein ☐
- Thüringen ☐

I4. Was beschreibt Ihr Arbeitsumfeld am nächsten?

- Landgemeinde (< 5.000 Einwohner) ☐
- Kleinstadt (5001 - 20.000 Einwohner) ☐
- Einzugsgebiet einer Stadt ☐
- Großstadt (> 100.000 Einwohner) ☐

I5. **Was beschreibt Ihren überwiegenden Arbeitsplatz (> 50% Ihrer Arbeitszeit) am nächsten?**

- Krankenhaus ☐
- Universitätsklinikum ☐
- Rehabilitationseinrichtung ☐
- freie Praxis ☐
- Alters- oder Pflegeheim ☐

I6. **In welchem Arbeitsverhältnis stehen Sie überwiegend (> 50% Ihrer Arbeitszeit)?**

- Arbeitnehmer*In ☐
- Selbstständig ☐
- Freiberuflich ☐

I7. **Sind Sie in einer leitenden Funktion tätig?**

- Ja ☐
- Nein ☐

I8. **Arbeiten Sie in einem multiprofessionellen Team?**

- Ja ☐
- Nein ☐

I9. **Wie viele Jahre Berufserfahrung haben Sie?**

I10. **Wie viele Patient*Innen mit einem Schlaganfall behandeln Sie an einem üblichen Arbeitstag?**

- ≤ 5 ☐
- 6 - 10 ☐
- > 10 ☐

I11. **Wie viele Stunden arbeiten Sie normalerweise pro Woche als Physiotherapeut*In?**

LimeSurvey

I12. Welches ist Ihr höchster akademischer Abschluss?

- Bachelor ☐
- Diplom ☐
- Master ☐
- Promotion ☐
- Keiner ☐

I13. Wie viele Minuten Behandlungszeit stehen Ihnen für einen Patienten mit einem Schlaganfall zur Verfügung?

- ≤ 20 Minuten ☐
- 21 - 30 Minuten ☐
- 31 - 45 Minuten ☐
- > 45 Minuten ☐

Teil J: Teilnahme an weiterführenden Studien

J1. Für weiterführende Untersuchungen stehe ich Ihnen gern zur Verfügung. Bitte nutzen Sie dazu die im Kommentarfeld angegebene E-Mail-Adresse:

- Ja ☐
- Nein ☐

Teil K: Ergänzungen zum Fragebogen

K1. Folgende weitere Gedanken möchte ich zur Befragung beitragen:

LimeSurvey

Die Befragung ist nun beendet.

Ich bedanke mich vielmals für Ihre Teilnahme an dieser sowie an weiteren Untersuchungen und verbleibe

Mit freundlichen Grüßen,

Bettina Scheffler

Bitte kontaktieren Sie mich bei Fragen oder Anliegen:

Brandenburgische Technische Universität Cottbus – Senftenberg Fakultät 4/ Institut für Gesundheit Therapiewissenschaft I Wissenschaftliche Mitarbeiterin Bettina Scheffler Gebäude 1, Raum 1.302 Universitätsplatz 1 01968 Senftenberg T +49 (0)3573 85 707 E Bettina.Scheffler@b-tu.de

www.b-tu.de
