# Supplementary material for: Current physical therapy practice and implementation factors regarding the evidence-based ‘Rehabilitation of Mobility after Stroke (ReMoS)’ guideline in Germany: a cross-sectional online survey
Source: BMC Neurol. 2022 Jul 30;22:284. doi: 10.1186/s12883-022-02780-5 (PMC9338587; doi:10.1186/s12883-022-02780-5)
Supplement: Supplementary file 3 — Additional file 3. Translated English version of the questionnaire. [file 12883_2022_2780_MOESM3_ESM.pdf]

*Please note: This English language version is a direct translation of the original questionnaire without any form of cultural adaptation*

Title: Survey on physical therapy management to improve mobility of people after stroke in Germany.

Dear physical therapists,

Physical therapy interventions promote the mobility of people after a stroke.

In this nationwide survey, we assess which interventions physical therapists choose to improve walking ability, walking speed, walking distance, and balance in the subacute phase after stroke. We aim to evaluate the current physical therapy management of patients after stroke in Germany with regard to the recommendations of the S2e guideline "Rehabilitation of Mobility after Stroke (ReMoS)" and are interested which factors influence the implementation of the guideline recommendations. The results may help to understand whether people experience guidelines-based therapy after stroke and how physical therapists make decisions about the course of therapy.

<u>Survey Instructions</u>

We are asking you to answer the questions regarding the current physical therapy management for people in the subacute phase after stroke and some general demographic and occupational information. The questionnaire has a maximum of 89 questions and will require approximately 15-20 minutes of your time. Please answer the questions according to your current practice in rehabilitation of people after stroke.

Please read the explanations of terms that are attached to the questions as help texts. These explanations will help you to understand and answer the questions correctly. Use the opportunity to expand your answers with free text.

In addition, we would appreciate it if you would agree to participate in further surveys related to this research project and leave your email address for this purpose.

Thank you for supporting us by taking part in this research project!

You are welcome to invite your colleagues to participate and to forward the link to the survey.

If they have any questions or concerns, please feel free to contact:

Brandenburg University of Technology Cottbus-Senftenberg

Faculty 4 / Institute of Health

Therapy Sciences I

Research Assistant

Bettina Scheffler

Building 1, Room 1.302

Universitätsplatz 1

01968 Senftenberg

T +49 (0)3573 85 707

E Bettina.Scheffler@b-tu.de

www.b-tu.de

Confirmation of access requirements

Please confirm access requirements, if applicable, before beginning the voluntary survey.

**Are you a qualified physical therapist currently treating people in the subacute phase after a stroke in Germany?**

Please select one of the following answers:

Explanation: The subacute phase after a stroke refers to the time from the 4th week to the 6th month after stroke.

- o Yes
- o No

*(Note: If this question was answered with "No", the participants were redirected to the closing page.)*

**Section I**

**Interventions to improve the walking ability of non-ambulatory patients.**

How often do you use the following interventions to achieve walking ability in non-ambulatory people after a stroke?

<u>Intensive walking training</u>

Please select one of the following answers

Explanation: Intensive gait training means practicing individual gait phases or the entire gait cycle on the floor at an intensity of at least 30 minutes a day.

- o Always
- o Often
- o Sometimes
- o Never
- o No Answer

<u>Intensive walking training using end-effector-based device (e.g., gait trainers).</u>

Please select one of the following answers

Explanation: Intensive gait training means practicing individual gait phases or the entire gait cycle on the floor at an intensity of at least 30 minutes a day. Gait trainers are electromechanical end-effector-based training devices that use movable foot plates to train the gait cycle. Patients are supported and secured by harness systems.

- o Always
- o Often

- Sometimes
- Never
- No Answer

Intensive walking training using treadmill or exoskeleton (e.g. Lokomat).

Please select one of the following answers

Explanation: Intensive gait training means practicing individual gait phases or the entire gait cycle on the floor at an intensity of at least 30 minutes per day. Lokomats are exoskeleton-assisted training devices for training the gait cycle with guidance of the hips and legs.

- Always
- Often
- Sometimes
- Never
- No Answer

Functional electric stimulation in supine position

Please select one of the following answers

Explanation: Cyclic multichannel stimulation refers to a form of electrical stimulation to produce gait-like movements of the paretic leg hanging in the lateral position.

- Always
- Often
- Sometimes
- Never
- No Answer

To achieve ambulation in people after stroke who are unable to walk, I use other interventions. These are:

Please enter your answer:

**Interventions to improve the walking ability of patients with (partial) walking ability**

How often do you use the following interventions to improve the walking ability of (partially) ambulatory people after a stroke?

Intensive conventional walking training

Please select one of the following answers:

Explanation: Intensive conventional gait training means practicing individual gait phases or the entire gait cycle on the floor at an intensity of at least 30 minutes a day.

- Always
- Often
- Sometimes
- Never
- No Answer

Intensive progressive walking training using treadmill

Please select one of the following answers:

Explanation: Intensive gait training means practicing individual gait phases or the entire gait cycle at an intensity of at least 30 minutes a day. Progressive means continuously increasing the training demands by increasing the number of repetitions or increasing the difficulty of individual movement tasks.

- Always
- Often
- Sometimes
- Never
- No Answer

Task-specific training with motor imagery

Please select one of the following answers:

Explanation: Task-specific training means the improvement of movement execution by means of functional movement exercises. Motor imagery (mental training) is the mental imagination of movements of one's own body or body parts without actively executing them.

- Always
- Often
- Sometimes
- Never
- No Answer

Use of walking aids

Please select one of the following answers:

Explanation: Walking aids are devices used to directly or indirectly assist with gait training or balance, e.g., walking stick, ankle brace, weight cuffs, arm slings, taping.

- Always
- Often
- Sometimes
- Never
- No Answer

Combination therapy of end-effector-based device (e.g., gait trainer) with functional electrical stimulation.

Please select one of the following answers:

Explanation: Combination therapy means gait training using a gait trainer with simultaneous electrical stimulation of the quadriceps femoris muscle (for knee stabilization during the stance leg phase) and the peroneal nerve (activation of dorsal extension during the swing leg phase).

To improve the walking ability of (partially) ambulatory people after a stroke I use other interventions. These are:

Please enter your answer here:

**Interventions to improve the walking speed of (limited) ambulatory patients.**

How often do you use the following interventions to improve the walking speed of (limited) ambulatory people after a stroke?

Task-specific endurance training using treadmill

Please select one of the following answers:

Explanation: Task-specific training means the improvement of movement execution by means of functional movement exercises. Progressive means the continuous increase of the training demand by increasing the number of repetitions or increasing the difficulty of individual movement tasks.

- Always
- Often
- Sometimes
- Never
- No Answer

Task-specific endurance training using progressive circuit training

Please select one of the following answers:

Explanation: Task-specific training means the improvement of movement performance by means of functional movement exercises. Progressive means the continuous increase of the training demand by increasing the number of repetitions or increasing the difficulty of individual movement tasks.

- Always
- Often
- Sometimes
- Never
- No Answer

Intensive walking training without treadmill

Please select one of the following answers:

Explanation: Intensive gait training meant practicing individual gait phases or the entire gait cycle on the floor at an intensity of at least 30 minutes a day.

- Always
- Often
- Sometimes
- Never
- No Answer

Intensive walking training including a treadmill

Please select one of the following answers:

Explanation: Intensive gait training meant practicing individual gait phases or the entire gait cycle on the floor at an intensity of at least 30 minutes a day.

- Always
- Often
- Sometimes
- Never
- No Answer

Intensive, supervised, progressive home exercise program (strengthening, endurance, balance)

Please select one of the following answers:

Explanation: Intensive means training over several weeks. Supervised training means consultation and supervision of the exercise sessions by a physiotherapist at regular intervals. Progressive means the continuous increase of the training demand by increasing the number of repetitions or increasing the difficulty of individual movement tasks.

- Always
- Often
- Sometimes
- Never
- No Answer

Walking training with stimulation of flexor reflexes

Please select one of the following answers:

Explanation: Stimulation of flexor reflex afferents means sensory electrostimulation of the paretic leg with surface electrodes during walking. Thus, flexion synergies in the hip and

knee joints as well as dorsiflexion in the ankle joint are generated synchronously with the stride. This supports the swing leg phase in the gait cycle.

Additional functional leg training

Please select one of the following answers:

Explanation: Additional means the implementation of training in addition to standard therapy. Leg function training means specific training for the lower extremity.

- Always
- Often
- Sometimes
- Never
- No Answer

Intensive progressive task-specific training

Please select one of the following answers:

Explanation: Progressive means the continuous increase of the training demand by increasing the number of repetitions or increasing the difficulty of individual movement tasks. Task-specific training is the selection of movement exercises based on actual activities of daily living.

- Always
- Often
- Sometimes
- Never
- No Answer

Task-specific training with motor imagery

Please select one of the following answers:

Explanation: Task-specific training means the improvement of movement execution by means of functional movement exercises. Mental training means the mental imagination of movements of one's own body or body parts without actively executing them.

- Always
- Often
- Sometimes
- Never
- No Answer

Walking training using end-effector-based device (e.g., gait trainer) or gait training with an exoskeleton-supported device (e.g., Lokomat)

Please select one of the following answers:

Explanation: Gait trainers are electromechanical end-effector-based training devices that use movable foot plates to train the gait cycle. Patients are supported and secured by harness systems. Lokomats are exoskeleton-supported training devices for training the gait cycle with guidance of the hips and legs.

<u>Muscular endurance training</u>

Please select one of the following answers:

- Always
- Often
- Sometimes
- Never
- No Answer

<u>Isokinetic strength training</u>

Please select one of the following answers:

Explanation: Isokinetic strength training means the application of continuous resistance throughout the amplitude of the movement.

- Always
- Often
- Sometimes
- Never
- No Answer

<u>Walking training with acoustic stimulation</u>

Please select one of the following answers:

Explanation: Gait training means practicing individual gait phases or the entire gait cycle on the floor. Acoustic stimulation means an audible external clock (e.g., metronome) to improve motor control in movement sequences.

- Always
- Often
- Sometimes
- Never
- No Answer

<u>Acoustic feedback while walking</u>

Please select one of the following answers:

Explanation: Acoustic feedback means an audible signal as feedback on the quality or success of movement to optimize the learning process.

- Always
- Often
- Sometimes
- Never
- No Answer

Feedback / Reinforcement

Please select one of the following answers:

Explanation: Feedback and reinforcement mean specific, reinforcing verbal feedback on speed to optimize the learning process.

- Always
- Often
- Sometimes
- Never
- No Answer

Combination of end-effector-based device (e.g., gait trainer) with functional electrical stimulation

Please select one of the following answers:

Explanation: Combination therapy means gait training using a gait trainer with simultaneous electrical stimulation of the quadriceps femoris muscle (for knee stabilization during the stance leg phase) and the peroneal nerve (activation of dorsal extension during the swing leg phase).

- Always
- Often
- Sometimes
- Never
- No Answer

Early use of ankle-foot-orthoses

Please select one of the following answers:

Explanation: Ankle orthoses are used to correct malpositions of the foot due to weakness of the foot lever, thus enabling a firmer hold when walking.

- Always
- Often
- Sometimes
- Never
- No Answer

Early use of orthopedic shoes

Please select one of the following answers:

Explanation: Temporary use of orthopedic shoes at the beginning of rehabilitation.

- Always
- Often
- Sometimes
- Never
- No Answer

Use of arm slings for severe arm paresis

Please select one of the following answers:

- Always
- Often
- Sometimes
- Never
- No Answer

To improve the walking speed of (limited) ambulatory people after a stroke I use other interventions. These are:

Please enter your answer here:

**Interventions to improve walking distance of (limited) ambulatory patients.**

How often do you use the following interventions to extend the walking distance of (limited) ambulatory people after a stroke?

Task-specific endurance training

Please select one of the following answers:

Explanation: Task-specific training means the improvement of movement execution by means of functional movement exercises.

- Always
- Often
- Sometimes
- Never
- No Answer

Intensive, supervised, progressive home exercise program (strengthening, endurance, balance)

Please select one of the following answers:

Explanation: Supervised training means advice and supervision by a physical therapist during an exercise session. Progressive means the continuous increase of training demands by increasing the number of repetitions.

- Always
- Often
- Sometimes
- Never
- No Answer

Intensive walking training using treadmill; especially progressive aerobic treadmill training

Please select one of the following answers:

Explanation: Intensive gait training means practicing individual gait phases or the entire gait cycle at an intensity of at least 30 minutes per day. Progressive means continuously increasing the training demand by increasing the number of repetitions or increasing the difficulty of individual movement tasks. Aerobic training means according to the criteria of the American College of Sports Medicine (ACSM) and the American Heart Association/American Stroke Association (AHA/ASA) a 20 - 60 minute training of endurance on 3 to 7 days a week. Exercise intensity should be between 50-80% of maximal heart rate. Alternatively, the exertion should correspond to a subjective sensation of 11 to 14 points on a subjective exertion sensation scale (6-20 points).

- Always
- Often
- Sometimes
- Never
- No Answer

<u>Task-specific training with motor imagery</u>

Please select one of the following answers:

Explanation: Task-specific training means the improvement of movement execution by means of functional movement exercises. Motor imagery (mental training) is the mental imagination of movements of one's own body or body parts without actively executing them.

- Always
- Often
- Sometimes
- Never
- No Answer

<u>Walking training with an end-effector-based device (e.g., gait trainer) or gait training with an exoskeleton-supported gait trainer (e.g., Lokomat).</u>

Please select one of the following answers:

Explanation: Gait trainers are electromechanical end-effector-based training devices that use movable footplates to train the gait cycle. Lokomats are exoskeleton-supported training devices for training the gait cycle with guidance of the hips and legs.

- Always
- Often
- Sometimes
- Never
- No Answer

<u>Muscular endurance training</u>

Please select one of the following answers:

- o Always
- o Often
- o Sometimes
- o Never
- o No Answer

Functional electrical multichannel stimulation during walking training

Please select one of the following answers:

Explanation: Functional Electrical Multichannel Stimulation means the selective step-synchronous stimulation of several muscles/ muscle groups of the paretic leg during gait.

- o Always
- o Often
- o Sometimes
- o Never
- o No Answer

High-frequency physical therapy at home

Please select one of the following answers:

- o Always
- o Often
- o Sometimes
- o Never
- o No Answer

Additional leg function training

Please select one of the following answers:

Explanation: Leg function training means specific training for the lower extremity. Additional means the implementation of training in addition to standard therapy.

- o Always
- o Often
- o Sometimes
- o Never
- o No Answer

Additional muscular-endurance training

Please select one of the following answers:

Explanation: Additional means the performance of training in addition to regular therapy.

- o Always
- o Often
- o Sometimes

- Never
- No Answer

To extend the walking distance of (limited) ambulatory people after a stroke I use other interventions.

These are:

**Measures to prevent falls and improve static and dynamic balance**

How often do you use the following interventions to improve static and dynamic balance in people after stroke and prevent falls?

Intensive walking training without a treadmill

Please select one of the following answers:

Explanation: Intensive gait training means practicing individual gait phases or the entire gait cycle on the floor at an intensity of at least 30 minutes a day.

- Always
- Often
- Sometimes
- Never
- No Answer

Intensive walking training including the treadmill

Please select one of the following answers:

Explanation: Intensive gait training means practicing individual gait phases or the entire gait cycle on the treadmill at an intensity of at least 30 minutes a day.

- Always
- Often
- Sometimes
- Never
- No Answer

Intensive, supervised, progressive home exercise program (strengthening, endurance, balance)

Please select one of the following answers:

Explanation: Supervised training means advice and supervision by a physical therapist during an exercise session. Progressive means the continuous increase of the training demand by increasing the number of repetitions or increasing the difficulty of individual movement tasks.

- Always
- Often
- Sometimes
- Never
- No Answer

Motor Relearning Program

Please select one of the following answers:

Explanation: The Motor Relearning Program means task-oriented learning of activities of daily living to improve motor function.

- Always
- Often
- Sometimes
- Never
- No Answer

Walking training using a treadmill, an end-effector-based device (e.g., gait trainer), or an exoskeleton-supported device (e.g., Lokomat).

Please select one of the following answers:

Explanation: Gait trainers are electromechanical end-effector-based training devices that use movable foot plates to train the gait cycle. Patients are supported and secured by harness systems. Lokomats are exoskeleton-supported training devices for training the gait cycle with guidance of the hips and legs.

- Always
- Often
- Sometimes
- Never
- No Answer

Muscular endurance training

Please select one of the following answers:

- Always
- Often
- Sometimes
- Never
- No Answer

Trunk control training on unstable surface

Please select one of the following answers:

- Always
- Often
- Sometimes
- Never
- No Answer

Acoustic feedback while walking

Please select one of the following answers:

Explanation: Acoustic feedback means an audible external signal as feedback on the quality or success of movement.

- Always
- Often
- Sometimes
- Never
- No Answer

Early use of orthopedic shoes

Please select one of the following answers:

Explanation: Temporary use of orthopedic shoes at the beginning of rehabilitation.

- Always
- Often
- Sometimes
- Never
- No Answer

Additional ergometer training

Please select one of the following answers:

Explanation: Additional means the implementation of training in addition to the regular rehabilitation program.

- Always
- Often
- Sometimes
- Never
- No Answer

Additional training on a biofeedback platform with extended tasks

Please select one of the following answers:

Explanation: Additional means the implementation of training in addition to conventional therapy. Biofeedback platform means force plates that provide feedback on the position of the body's center of gravity via a monitor to support the learning process.

- Always
- Often
- Sometimes
- Never
- No Answer

<u>To improve static and dynamic balance of people after stroke and prevent falls I use other interventions. These are:</u>

Please enter your answer here:

**Section II**

S2-e guideline "Rehabilitation of mobility after stroke".

Thank you for answering the questions up to this point! Please answer now some questions about your personal experience with guidelines.

<u>I know the S2e guideline "Rehabilitation of mobility after stroke (ReMoS)".</u>

Please select one of the following answers:

- Yes
- No

*(Note: If this question was answered with "No", the participants were forwarded to Section III.)*

<u>I learned about the ReMoS guideline through the following channel:</u>

Please enter your answer here:

**Barriers and facilitators to implementing guideline recommendations.**

Please select the appropriate answers:

Fully agree, Agree, Do not agree nor disagree, Disagree, Fully disagree

- The ReMoS-Guideline leaves enough room for me to make my own conclusions.
- The ReMoS-Guideline leaves enough room to weigh the wishes of the patient.
- The ReMoS-Guideline is a good starting point for my self-study.
- I did not thoroughly read nor remember the ReMoS-Guideline.
- I wish to know more about the ReMoS-Guideline before I decide to use it.
- I have problems changing my old routines.

- I think parts of the ReMoS-Guideline are incorrect.
- I have a general resistance to working according to protocols.
- Fellow physical therapists do not cooperate in using the ReMoS-Guideline.
- Neurologists and other physicians do not cooperate in using the ReMoS-Guideline.
- Managers/directors do not cooperate in using ReMoS-Guideline.
- Patients do not cooperate in using the ReMoS-Guideline.
- Working according to the ReMoS-Guideline is too time consuming.
- The ReMoS-Guideline does not fit into my ways of working in daily practice.
- Working according to the ReMoS-Guideline requires financial compensation.
- The layout of the ReMoS-Guideline makes it handy for use.

In my opinion, other factors also condition the implementation of the ReMoS guideline. These are:

Please select the answers that apply:

- Availability of an end-effector-device (e.g., gait trainer).
- Availability of an exoskeleton-device (e.g., Lokomat)
- Availability of a treadmill
- Knowledge of recommended interventions
- Involvement of other professional groups (e.g. sports therapists, masseurs and medical bath attendants, ...)
- Lack of reference to the phase model of neurological rehabilitation
- Other

**Section III**

**Sociodemographic data**

The survey is almost finished. I would now like to ask you to provide information about yourself and your work as a physical therapist.

<u>How old are you?</u>

Only numbers may be entered in this field.

Your answer must be between 18 and 80.

<u>What is your gender?</u>

- Divers
- Female
- Male
- No answer

In which state do you work?

Please select one of the following answers:

- Baden-Württemberg
- Bayern
- Berlin
- Brandenburg
- Bremen
- Hamburg
- Hessen
- Mecklenburg-Vorpommern
- Niedersachsen
- Nordrhein-Westfalen
- Rheinland-Pfalz
- Saarland
- Sachsen
- Sachsen-Anhalt
- Schleswig-Holstein
- Thüringen
- No answer

What most closely describes your work environment?

Please select one of the following answers:

- Rural community (< 5,000 inhabitants)
- Small town (5001 - 20,000 inhabitants)
- Catchment area of a city
- Large city ( > 100,000 inhabitants)
- No answer

What most closely describes your predominant place of employment (> 50% of your working hours)?

Please select one of the following answers:

- Hospital
- University hospital
- Rehabilitation facility
- independent practice
- nursing home
- No answer

In which employment relationship are you predominantly employed (> 50% of your working hours)?

Please select one of the following answers:

- Employee
- Self-employed
- Freelance
- No answer

<u>Do you work in a leadership/ management position?</u>

Please select one of the following answers:

- Yes
- No
- No answer

<u>Do you work in a multi-professional team?</u>

Please select one of the following answers:

- Yes
- No
- No answer

<u>How many years of professional experience do you have?</u>

Only numbers may be entered in this field.

<u>How many patients* with a stroke do you treat in a usual working day?</u>

Please select one of the following answers:

- ≤ 5
- 6 - 10
- 10
- No answer

<u>How many hours do you usually work per week as a physical therapist*?</u>

Only numbers may be entered in this field.

Your answer must be between 1 and 48.

<u>What is your highest academic degree?</u>

Please select one of the following answers:

- Bachelor's degree
- Diploma
- Master

- Doctorate
- None
- No answer

How many minutes of treatment time do you have for a patient with a stroke?

Please select one of the following answers:

- ≤ 20 minutes
- 21 - 30 minutes
- 31 - 45 minutes
- 45 minutes
- No answer

Participation in further studies

For further studies I am at your disposal. Please use the email address provided in the comments field:

Please select one of the following answers:

- Yes
- No
- No answer

Please enter your e-mail address here:

Additional comments to the questionnaire

I would like to contribute the following additional thoughts to the questionnaire:

The survey is now closed.

Thank you very much for your participation in this and in further surveys.

Yours sincerely,

Bettina Scheffler

Please contact me with any questions or concerns:

Brandenburg University of Technology Cottbus - Senftenberg

Faculty 4/ Institute of Health

Therapy Science I

Research Assistant

Bettina Scheffler

Building 1, Room 1.302

University Place 1

01968 Senftenberg

T +49 (0)3573 85 707

E Bettina.Scheffler@b-tu.de

www.b-tu.de
