# Supplementary material for: Current physical therapy practice and implementation factors regarding the evidence-based ‘Rehabilitation of Mobility after Stroke (ReMoS)’ guideline in Germany: a cross-sectional online survey
Source: BMC Neurol. 2022 Jul 30;22:284. doi: 10.1186/s12883-022-02780-5 (PMC9338587; doi:10.1186/s12883-022-02780-5)
Supplement: Supplementary file 4 — Additional file 4. Responses on open-ended questions according therapy-goal directed interventions within ReMoS guideline domains. [file 12883_2022_2780_MOESM4_ESM.pdf]

Additional file 3.

Responses on open-ended questions according therapy-goal directed interventions within ReMoS guideline domains.

**Improving ability to walk in non-ambulant patients**	**n**	**Improving ability to walk in (partially) ambulant patients**	**n**	**Improving walking distance**	**n**	**Improving walking speed**	**n**	**Improving balance**	**n**
Concept-based interventions	22	Concept-based interventions	8	Task-specific gait training	3	Concept-based interventions	8	Balance training using modified supporting area	10
Functional training	10	Strength training	3	Forms of endurance training	3	Ergometer training (including water-based)	1	Concept-based interventions	7
Gait training using walking aids	10	Trunk training (mobilisation and stabilisation)	3	Gait training using treadmill	2	Feedback on walking distance	1	Task-specific balance training	4
Training of activities of daily living	5	Gait training using treadmill	2	Concept-based interventions	2	Hippotherapy	1	Balance training using destabilising impulses on the body	3

Balance training with and without therapy devices	4	Gait training using walking aids	2	Respiratory therapy	1	Co-ordination training	1	Bicycle ergometer training	1
Leg trainer (Motomed)	3	Balance training	2	Conventional gait training	1	Neuromobilization	1	Strength training	1
Strength training	3	Stairs training	2			Trunk training	1	Patient education	1
Conventional gait training	3	Movement exercise with visual control	1			Mental training (mirror therapy)	1	Sensitivity training	1
Trunk training (mobilisation and stabilisation)	3	Electro-mechanical assisted gait training	1			Medical Taping	1		

Movement therapy in lying position	2	Additional fitness training	1
Foot orthosis	2	Functional electrostimulation in combination with exoskeleton	1
Electro-mechanical assisted gait training	1	Gait training with acoustic stimulation (music)	1
Functional electrostimulation	1	Fall training	1
Functional electrostimulation in combination with exoskeleton	1	Medical taping	1

Movement therapy using sling table	1
Group exercise	1
Mental training (imaginary journeys)	1
Medical taping	1
Training of sensory-motor perception	1
